# Supplementary material for: Synapsin-caveolin-1 gene therapy preserves neuronal and synaptic morphology and prevents neurodegeneration in a mouse model of AD
Source: Mol Ther Methods Clin Dev. 2021 Mar 29;21:434–50. doi: 10.1016/j.omtm.2021.03.021 (PMC8065227; doi:10.1016/j.omtm.2021.03.021)
Supplement: Document S1. Figures S1–S7 [file mmc1.pdf]

OMTM, Volume *21*

Supplemental information

Synapsin-caveolin-1 gene therapy preserves neuronal and synaptic morphology and prevents neurodegeneration in a mouse model of AD

Shanshan Wang, Joseph S. Leem, Sonia Podvin, Vivian Hook, Natalia Kleschevnikov, Paul Savchenko, Mehul Dhanani, Kimberly Zhou, Isabella C. Kelly, Tong Zhang, Atsushi Miyanohara, Phuong Nguyen, Alexander Kleschevnikov, Steve L. Wagner, John Q. Trojanowski, David M. Roth, Hemal H. Patel, Piyush M. Patel, and Brian P. Head

Supplementary Materials

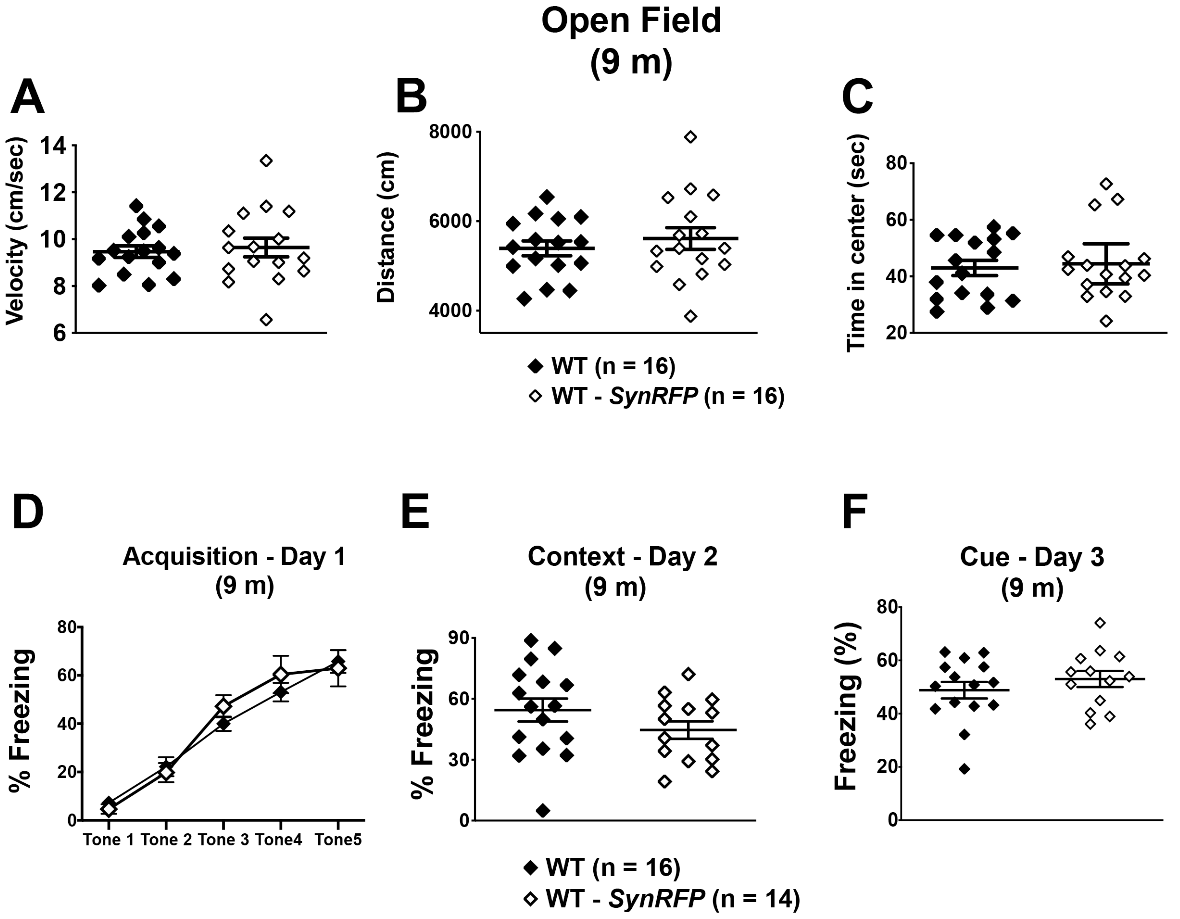

Supplemental Figure 1 Open field and fear conditioning test comparing naïve WT with WT-*SynRFP* at 9 m. (**A-C**) Open field (velocity, distance moved, time in center) and (**D-F**) fear conditioning for naïve WT (n = 16) versus WT-*SynRFP* (n =14-16) at 9 m. Open field data (mean ± SEM) (percent (%) freezing mean ± SEM) were analyzed using Student t test (Day 2 and 3). Significance was assumed when $p < 0.05$.

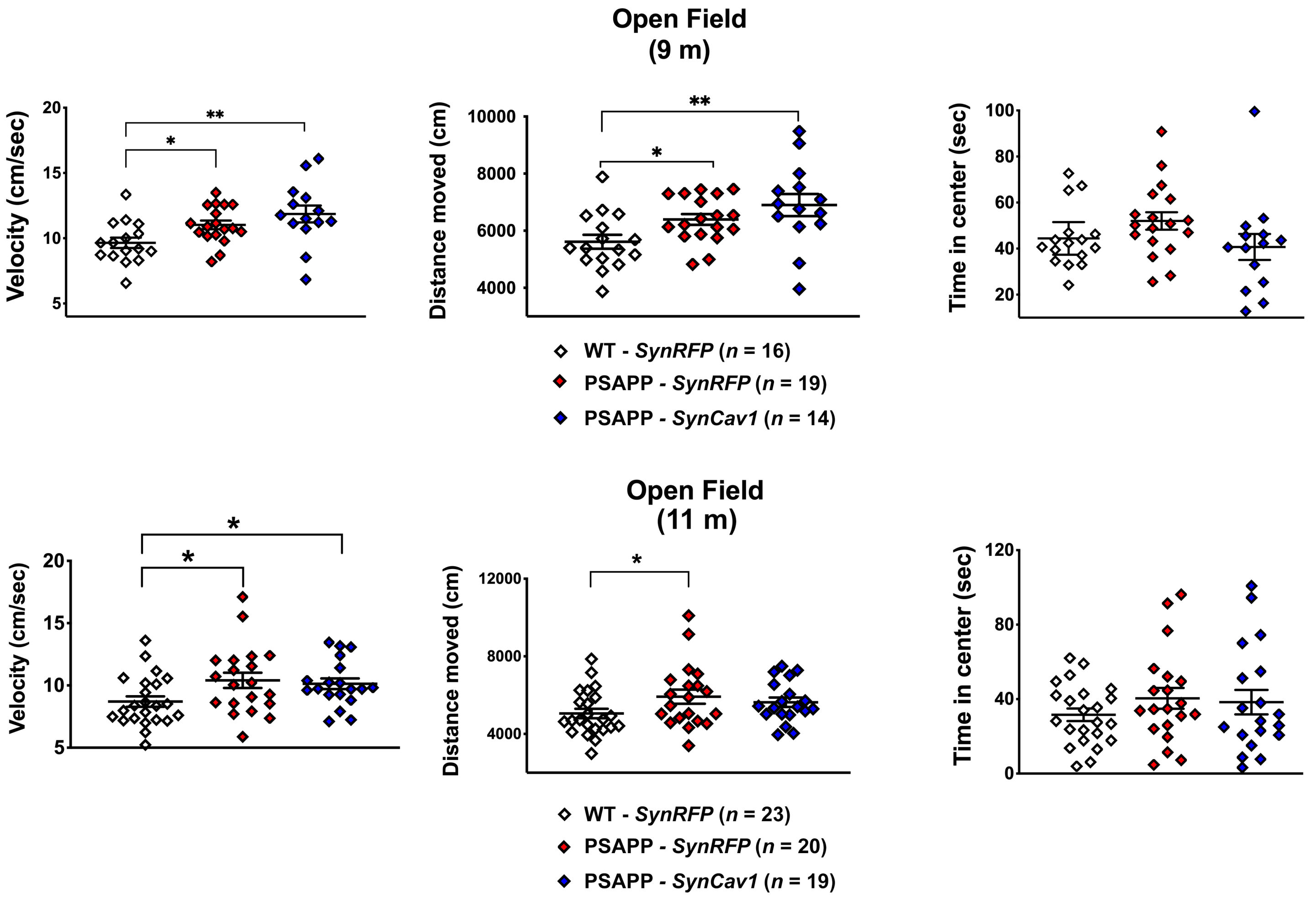

Supplemental Figure 2 Open field performance of 9 and 11 m PSAPP *(APPSwePS1d9*) mice. (A**, D**) Mean velocity, (**B, E**) distance traveled, and (**C, F**) time spent in the center of 9 and 11 m old WT-*SynRFP* (n = 16 at 9 m; n = 23 at 11 m), PSAPP-*SynRFP* (n = 19 at 9 m; n = 20 at 11 m), and PSAPP-*SynCav1* (n = 14 at 9 m; n = 19 at 11 m) mice respectively. For open field data (mean ± SEM) was analyzed using one-way analysis of variance (ANOVA) followed by Fisher's LSD multiple comparisons tests. Data are presented as mean ± SEM. Significance was assumed when $p < 0.05$. *$p < 0.05$, **$p < 0.01$.

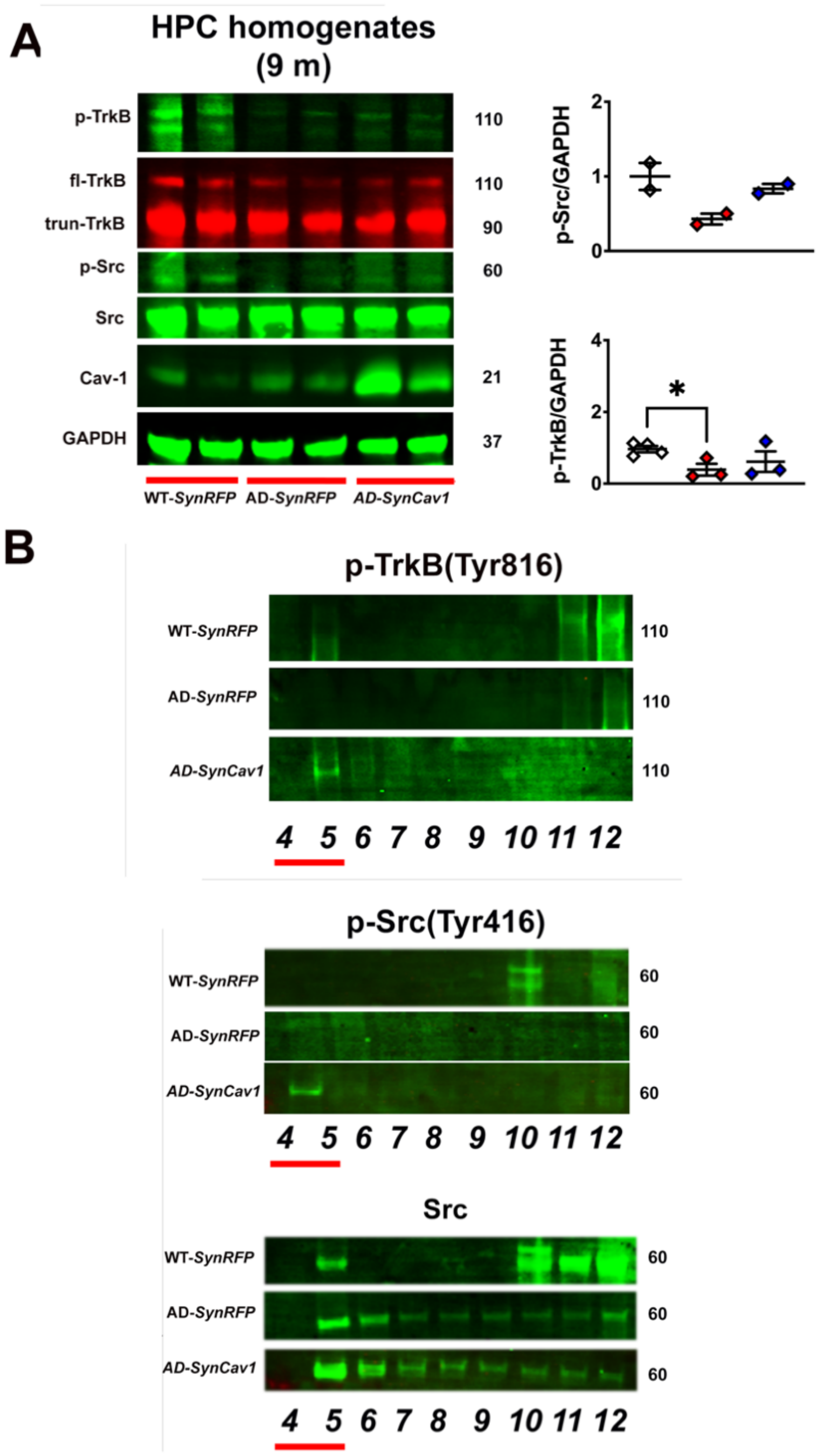

Supplemental Figure 3 Assessment of TrkB activation in hippocampal homogenates and MLR fractions at 9 m. (**A**) Hippocampal homogenates from 9 m old WT-*SynRFP,* AD-*SynRFP*, and AD-*SynCav1* mice (n=3) were assayed for phosphorylated (p-)TrkB (Y816), full length (fl) and truncated (trun) TrkB, p-Src (Y416), Src, Cav-1, and GAPDH. (**B**) Fractions were assayed for p-TrkB (Y816) and p-Src (Y418). Fractions were generated

from equal protein (0.5 ug/ul). Data (mean ± SEM) were analyzed using one-way ANOVA. Significance was assumed when *$p < 0.05$.

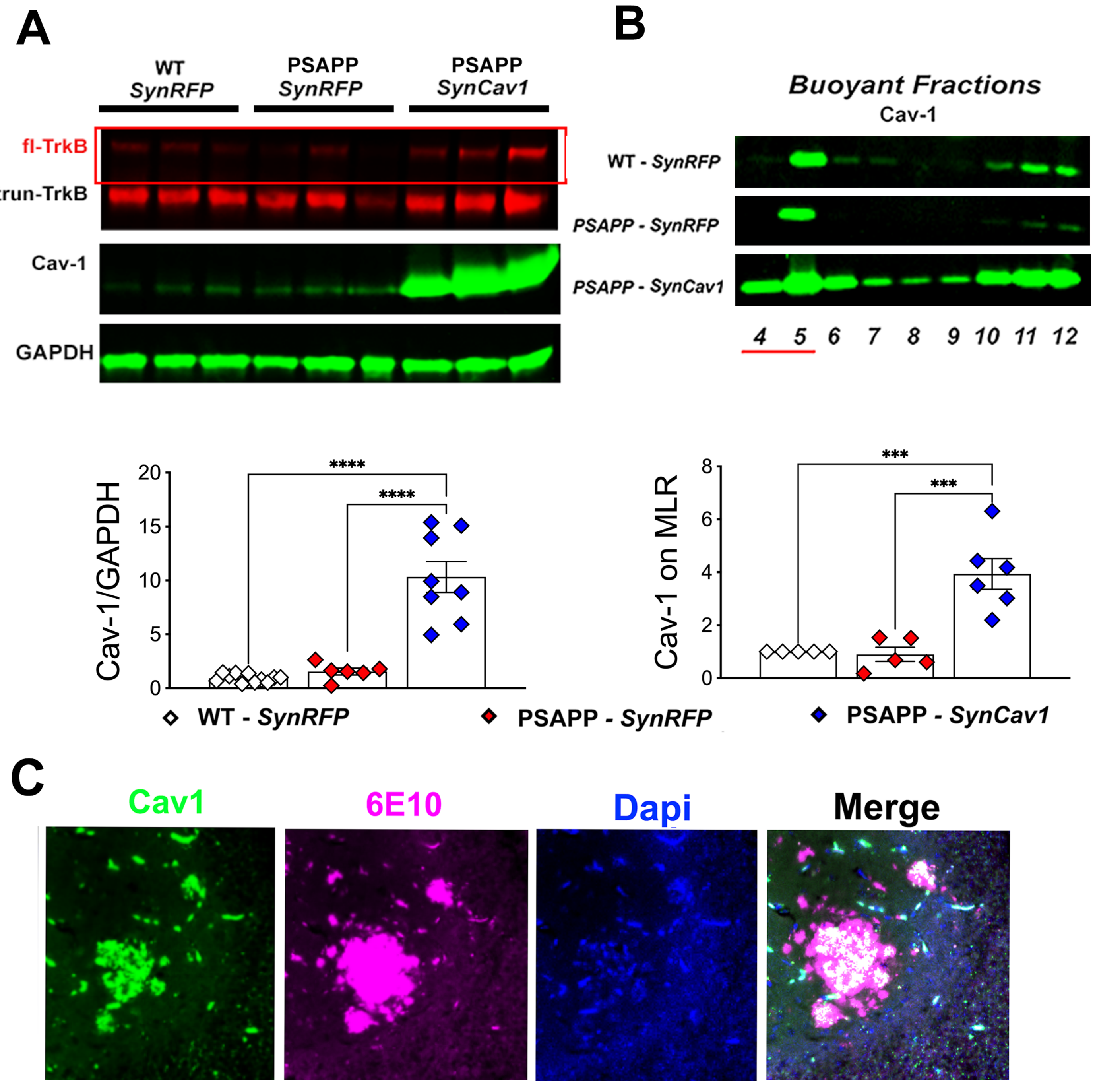

Supplemental Figure 4 (A) IB of HPC homogenates and **(B)** MLR fractions confirm the sustained overexpression of Cav1 in mice hippocampi. **(C)** Colocalization of Cav-1 and 6E10 was observed in 11-month

old PSAPP-SynRFP mice. Data (mean ± SEM) were analyzed using one-way ANOVA followed by Fisher's LSD multiple comparisons test. Significance was assumed when $*p < 0.05$.

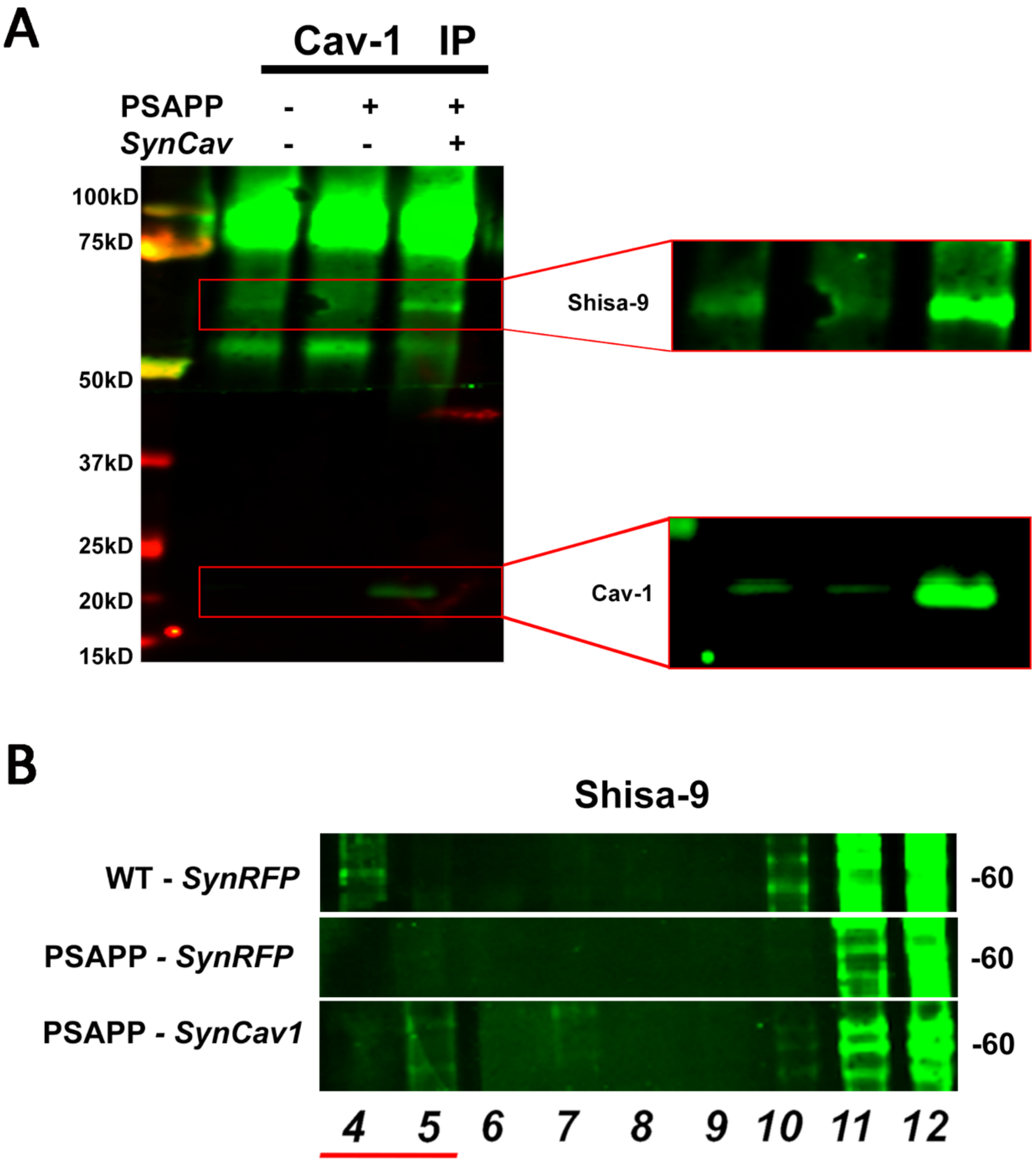

Supplemental Figure 5 Cav-1 IPs of MLR fractions confirm the interaction of Shisa9 and Cav1 in mice hippocampi **(A)**. Cav-1 IP of MLR fractions revealed decreased Shisa9 expression in PSAPP -SynRFP and the highest Shisa9 expression in PSAPP-*SynCav1*. **(B)** Representative blot of Shisa9 in hippocampal MLR fractions in WT-*SynRFP*, PSAPP-*SynRFP*, and PSAPP-*SynCav1* mice at 9 m.

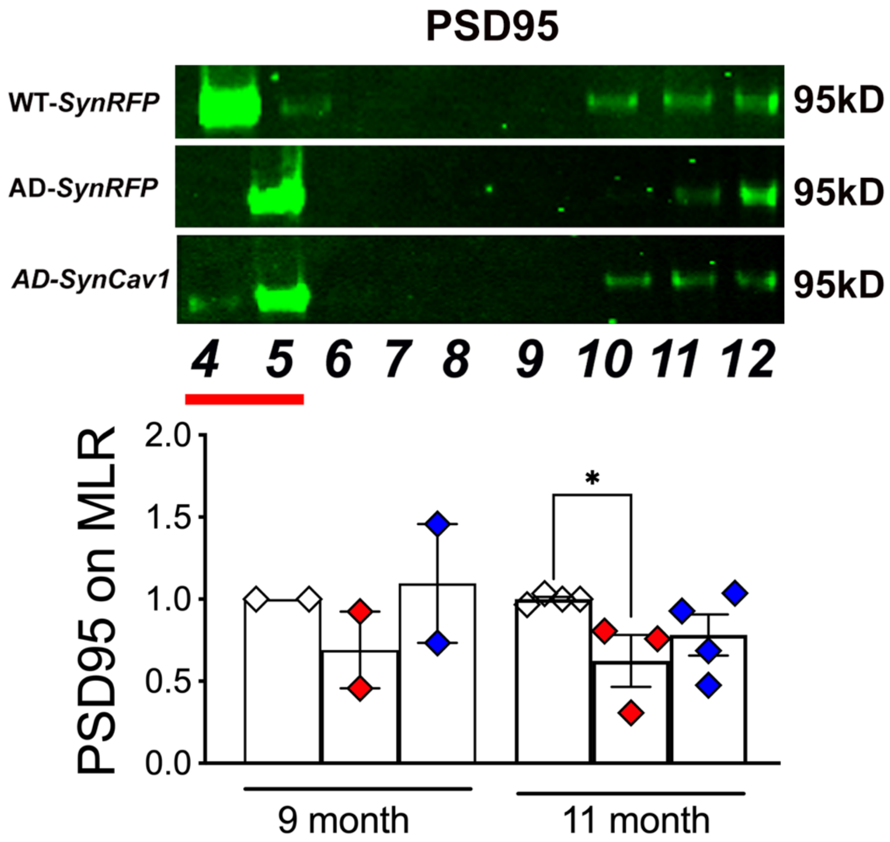

Supplemental Figure 6 Immunoblots of MLR fractions revealed a decrease of PSD in 11 month PSAPP-*SynRFP*. Immunoblot is representative of 11-month samples. No significant difference was detected between AD-*SynCav1* and WT-*SynRFP* mice at 11 m.

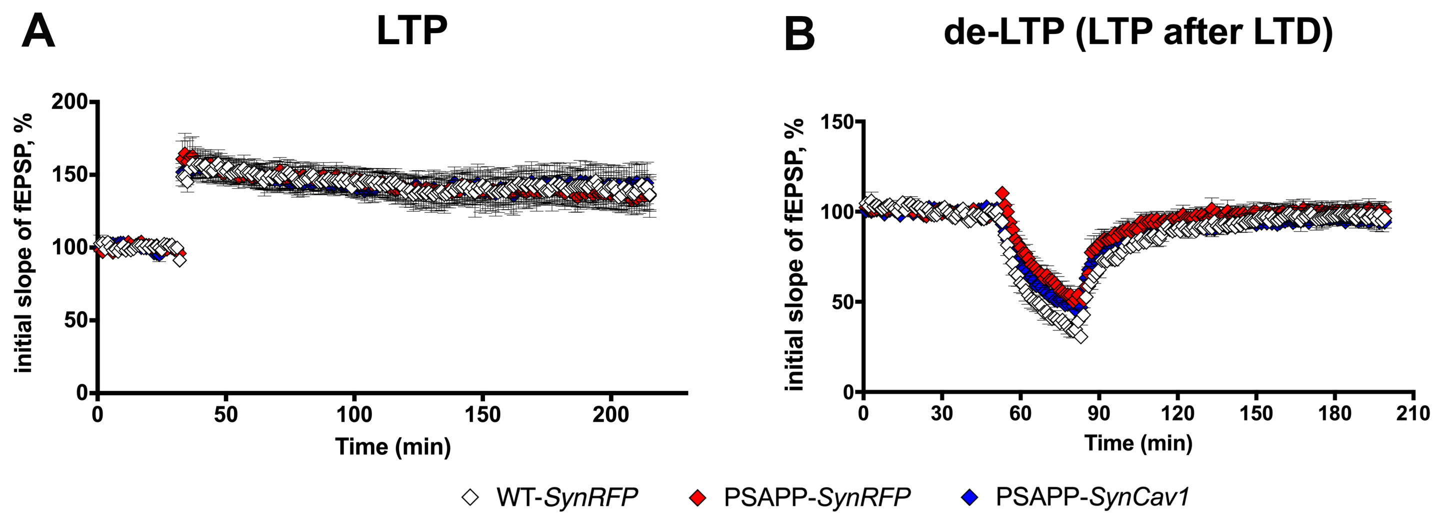

Supplemental Figure 7 Electrophysiology reveals no significant difference in long-term potentiation (LTP) between WT-*SynRFP*, PSAPP-*SynRFP*, and PSAPP-*SynCav1* hippocampal slices (**A**). De-LTP (LTD after prior induction of LTP) was evoked by low-frequency stimulation (LFS, 15 min x 1 Hz) applied 3 hours after induction of LTP. The short-term depression (1-5 min) revealed no differences among the groups. Long-term changes (1-2 hours after LFS) were also not different among the groups. Data (mean ± SEM) were analyzed using two-way analysis of variance (ANOVA), *n* = 7-8 slices from 3-4 mice/group.

Supplementary Video. 1 Light Sheet Microscopy AAV9-*SynRFP* injected mouse hippocampus (1-week post-injection), optically cleared in X-CLARITY hydrogel solution (Cat #13103). Data processing and 3D rendering was done using Arivis Vision4D™. Scale bar = 100 um
