# Supplementary material for: Synapsin-caveolin-1 gene therapy preserves neuronal and synaptic morphology and prevents neurodegeneration in a mouse model of AD
Source: Mol Ther Methods Clin Dev. 2021 Mar 29;21:434–50. doi: 10.1016/j.omtm.2021.03.021 (PMC8065227; doi:10.1016/j.omtm.2021.03.021)
Supplement: Document S2. Article plus supplemental information [file mmc3.pdf]

Molecular Therapy
Methods & Clinical Development
Original Article

# Synapsin-caveolin-1 gene therapy preserves neuronal and synaptic morphology and prevents neurodegeneration in a mouse model of AD

Shanshan Wang,[1,2] Joseph S. Leem,[1,2] Sonia Podvin,[4] Vivian Hook,[4] Natalia Kleschevnikov,[1,2] Paul Savchenko,[2] Mehul Dhanani,[1,2] Kimberly Zhou,[1,2] Isabella C. Kelly,[2] Tong Zhang,[6] Atsushi Miyanohara,[1,2] Phuong Nguyen,[3] Alexander Kleschevnikov,[3] Steve L. Wagner,[1,3] John Q. Trojanowski,[5] David M. Roth,[1,2] Hemal H. Patel,[1,2] Piyush M. Patel,[1,2] and Brian P. Head[1,2]

[1]Veterans Affairs San Diego Healthcare System, San Diego, CA, USA; [2]Department of Anesthesiology, University of California San Diego, San Diego, CA 92161, USA; [3]Department of Neurosciences, University of California San Diego, La Jolla, CA, USA; [4]Skaggs School of Pharmacy and Pharmaceutical Sciences, University of California San Diego, La Jolla, CA, USA; [5]Center for Neurodegenerative Disease Research, Department of Pathology and Laboratory Medicine, Perelman School of Medicine at the University of Pennsylvania, Philadelphia, PA 19104-4283, USA; [6]Campus Microscopy & Imaging Facility (CMIF)/Microscopy Shared Resource (MSR), The Ohio State University, OH, USA

**Alzheimer's disease (AD) is the most common form of neurodegeneration and cognitive dysfunction in the elderly. Identifying molecular signals that mitigate and reverse neurodegeneration in AD may be exploited therapeutically. Transgenic AD mice (PSAPP) exhibit learning and memory deficits at 9 and 11 months, respectively, with associated decreased expression of caveolin-1 (Cav-1), a membrane/lipid raft (MLR) scaffolding protein necessary for synaptic and neuroplasticity. Neuronal-targeted gene therapy using synapsin-Cav-1 cDNA (*SynCav1*) was delivered to the hippocampus of PSAPP mice at 3 months using adeno-associated virus serotype 9 (AAV9). Bilateral *SynCav1* gene therapy was able to preserve MLRs profile, learning and memory, hippocampal dendritic arbor, synaptic ultrastructure, and axonal myelin content in 9- and 11-month PSAPP mice, independent of reducing toxic amyloid deposits and astrogliosis. Our data indicate that *SynCav1* gene therapy may be an option for AD and potentially in other forms of neurodegeneration of unknown etiology.**

## INTRODUCTION

Alzheimer's disease (AD) is a devastating neurodegenerative condition and the most common cause of dementia. Although AD is characterized by abundant amyloid-β (Aβ) plaques and neurofibrillary tau tangles, disrupted synaptic signaling and loss of synapses and neurons are more closely correlated to cognitive deficits. Currently, available interventions only ease the symptoms, suggesting that removal of toxic amyloid species alone may not be sufficient to reverse functional deficits in the AD brain. We thus postulate that in order to combat AD, a combination of approaches that promote neuronal and synaptic plasticity while simultaneously decreasing amyloid plaques may work in concert to attenuate or reverse the neurodegenerative process. Gene therapies that target neuroprotection and resilience may be an effective option to treat individuals afflicted with AD or other forms of neurodegeneration of different or unknown etiology.[1,2]

Cellular plasma membranes contain discrete microdomains enriched in synaptic signaling components and cholesterol termed membrane/lipid rafts (MLRs). Evidence shows that toxic Ab species aggregate with membrane cholesterol and other lipids resulting in damage or loss of MLR-related signaling proteins, ultimately leading to synaptic loss and cognitive dysfunction in AD.[3,4] One potential alternative to treat AD and other neurodegenerative conditions is the use of gene therapies that target neuroprotective mechanisms via restoration of MLR-localized functional neurotrophin receptors (NTRs).[5,6] Enhancing MLR-localization of NTRs may augment pro-survival and pro-growth signaling, enhance structural and functional neuroplasticity, or even increase the efficacy of exogenous or already present endogenous growth factors, which in turn may restore cognitive function in AD. One such gene therapy candidate is caveolin-1 (Cav-1), an MLR scaffolding protein that organizes NTRs (Trk) and neurotransmitter receptor signaling complexes in MLRs.[7–9] Both pre-clinical and clinical findings revealed that Cav-1 and Cav-1 associated signaling complexes (NTRs and neurotransmitter receptors) were decreased in degenerating neurons in AD, chronic traumatic encephalopathy (CTE), and amyotrophic lateral sclerosis (ALS).[10–13] In contrast, we previously showed that neuron-targeted Cav-1 overexpression (i.e., synapsin-promoted Cav-1 or *SynCav1*) augmented agonist-mediated synaptic signaling (e.g., NTRs, neurotransmitter receptors) and dendritic arborization *in vitro*.[7] Furthermore, we have shown that *SynCav1* transgenic (Tg) mice are resilient to traumatic

Received 17 November 2020; accepted 24 March 2021;
https://doi.org/10.1016/j.omtm.2021.03.021.

**Correspondence:** Brian P. Head, MS, PhD, Department of Anesthesiology, University of California San Diego, San Diego, CA 92161, USA.
**E-mail:** bhead@health.ucsd.edu


This is an open access article under the CC BY license (http://creativecommons.org/licenses/by/4.0/).

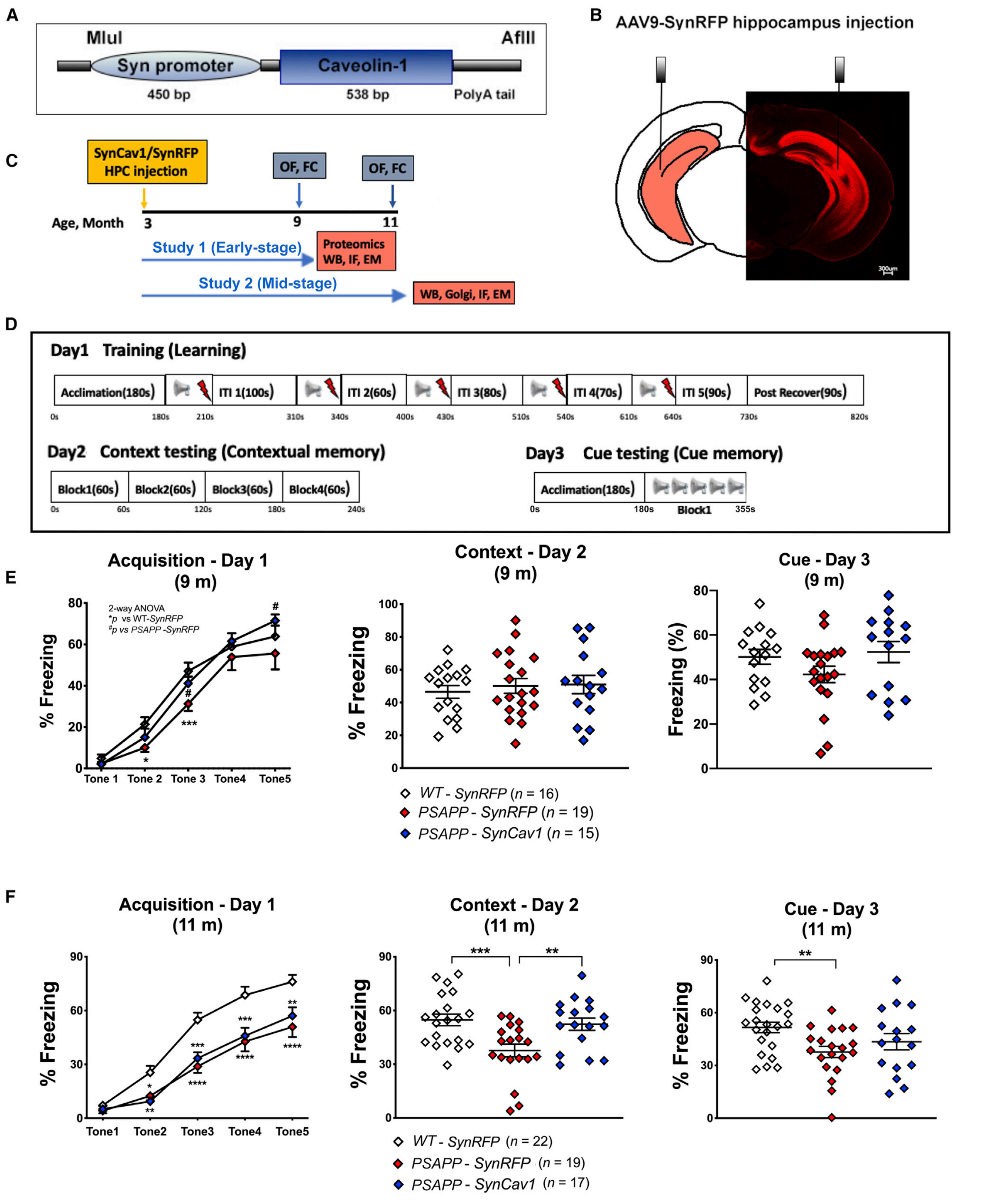


*(legend on next page)*

brain injury (TBI) and ALS, two neurodegenerative diseases of different etiology,[14] suggesting that Cav-1 may serve as a central neuroprotective target in a variety of neurodegenerative conditions.

The present study tested whether *SynCav1* gene therapy to Tg AD mice (PSAPP) could preserve MLR-localized fl-TrkB, preserve neuronal and synaptic plasticity, and improve higher brain function. These findings are the first to demonstrate that a one-time hippocampal delivery of *AAV-SynCav1* to PSAPP mice preserved hippocampal learning at 9 months and preserved memory at 11 months. Moreover, *SynCav1* preserved MLR-localization of full-length (fl)-TrkB, hippocampal dendritic arborization, synaptic ultrastructure, and axon myelin content. Thus, *SynCav1* gene therapy is an attractive approach to restore brain plasticity and improve higher brain function in AD and potentially in other forms of neurodegeneration caused by unknown etiology.

## RESULTS

### *SynCav1* preserves fear learning and memory in 9- and 11-month PSAPP mice

We first tested whether direct hippocampal delivery of AAV9 vectors would affect cognitive performance. No significant difference was observed in open field or fear conditioning behavioral tests between naive wild-type (WT) mice versus WT-*SynRFP* (Figure S1). Because there was no effect from AAV vectors on behavior, we did not include a naive WT control or naive PSAPP group (i.e., non-AAV) in this study. Previous work from our group demonstrated augmented functional neuroplasticity and improved hippocampal-dependent memory in WT mice that received AAV9-*SynCav1*;[8,15] therefore, to reduce experimental redundancy, the WT-*SynCav1* group was not included in the present study. To test the hypothesis that *SynCav1* can improve brain function in a neurodegenerative PSAPP mouse model, the current study utilized the following 3 groups to test this hypothesis: WT-*SynRFP*, PSAPP-*SynRFP*, and PSAPP-*SynCav1*.

Open field at 9 and 11 months revealed no general motor deficits nor anxiety-like behavior among the 3 groups. Both PSAPP-*SynRFP* and PSAPP-*SynCav1* showed hyperlocomotion compared to WT-*SynRFP* at 9 and 11 months, with no significant difference among two AD groups (Figure S2). At 9 months, PSAPP-*SynRFP* mice exhibited a significant reduction in fear learning acquisition on day 1 compared to WT-*SynRFP* (Figure 1E). In contrast, 9-month PSAPP-*SynCav1* mice exhibited preserved fear learning on day 1 (no difference versus WT-*SynRFP*). No significant difference in contextual (day 2) or cued memory recall (day 3) was observed among the groups. These results demonstrate that *SynCav1* gene delivery preserves fear learning in mild symptomatic PSAPP mice.

At 11 months, both PSAPP groups exhibited a significant reduction in learning on day 1 compared to WT-*SynRFP* (Figure 1F). On day 2, PSAPP-*SynRFP* mice exhibited a significant reduction in contextual memory recall versus WT-*SynRFP* and versus PSAPP-*SynCav1*, indicating hippocampal memory deficits. There was no significant difference between PSAPP-*SynCav1* versus WT-*SynRFP* on day 2 demonstrating preserved hippocampal-dependent memory recall in PSAPP mice that received *SynCav1*. On day 3, PSAPP-*SynRFP* mice exhibited a significant decrease in cue memory versus WT-*SynRFP*; no difference was observed between PSAPP-*SynCav1* versus WT-*SynRFP*. These data demonstrate direct evidence that *SynCav1* gene delivery preserves hippocampal memory in symptomatic PSAPP mice.

### *SynCav1* preserves MLR-localization of Cav-1 and TrkB in hippocampus of symptomatic PSAPP mice

Immunofluorescence (IF) of 9-month PSAPP-*SynRFP* mice showed decreased hippocampal Cav-1 expression, which was preserved in PSAPP-*SynCav1* (Figure 2A). Co-staining of the dendritic marker MAP2 with Cav-1 showed decreased MAP2 expression in the hippocampal CA1 subfield and cortex in PSAPP-*SynRFP*, similar to that observed in postmortem human brains of patients diagnosed with CTE, tauopathy, and AD.[12,13] In contrast, PSAPP-*SynCav1* mice displayed increased hippocampal (Figure 2B) and cortical (Figure 2C) MAP2 expression. Immunoblot (IB) assays of hippocampal homogenates confirmed decreased Cav-1 expression in PSAPP-*SynRFP* at 9 months (Figures 2D and 2E). Although no significant difference was found in brain homogenates between WT-*SynRFP* versus PSAPP-*SynRFP* in both full-length TrkB (fl-TrkB) and truncated TrkB (Trun-TrkB), PSAPP-*SynCav1* mice exhibited a significant increase in fl-TrkB versus PSAPP-*SynRFP* mice at 9 months, with no change in Trun-TrkB. IB of buoyant MLR fractions revealed a significant decrease in Cav-1, fl-TrkB, and Trun-TrkB in PSAPP-*SynRFP* versus WT-*SynRFP*. In contrast, MLRs from PSAPP-*SynCav1* mice exhibited a significant increase in Cav-1 and fl-TrkB (versus PSAPP-*SynRFP*; Figures 2D and 2E). Additional analysis comparing the ratio of fl-TrkB to Trun-TrkB revealed results that were similar to that detected with fl-TrkB alone. No changes in MLR-associated synaptobrevin or syntaxin expression (data not shown) were observed among the 3 groups, thus confirming the MLR changes were specific to Cav-1 and fl-TrkB. The decreased MLR-localized fl-TrkB expression in PSAPP-*SynRFP* mice indicates a subcellular alteration, which may in part contribute to neuronal dysfunction in AD; a subcellular alteration in PSAPP mice was prevented with *SynCav1*.

**Figure 1. *SynCav1* gene delivery preserves learning and memory in 9- and 11-month-old PSAPP (*APPSwePS1d9*) mice**

(A) Genetic construct of *SynCav1*. (B) Illustration (left) and microscopy of hippocampal RFP. (C) Schematic of experimental design in WT and PSAPP (*APPSwePS1d9*) mice. Blue arrows and rectangular boxes indicate open field and fear conditioning tests for separate 9- and 11-month cohorts; orange arrows and rectangular boxes indicate postmortem biochemical assays. (D) Schematic of fear conditioning protocol. (E and F) Fear conditioning test at 9 months (E) and at 11 months (F). Data are presented as percent (%) freezing mean ± SEM. Data were analyzed using two-way analysis of variance (ANOVA) followed by Fisher's LSD multiple comparisons tests (day 1) or one-way ANOVA (days 2 and 3). n = 15–19 animals per group for 9 months; n = 17–22 per group for 11 months. Significance was assumed when $p < 0.05$. *$p < 0.05$, **$p < 0.01$, ***$p < 0.001$, ****$p < 0.0001$.

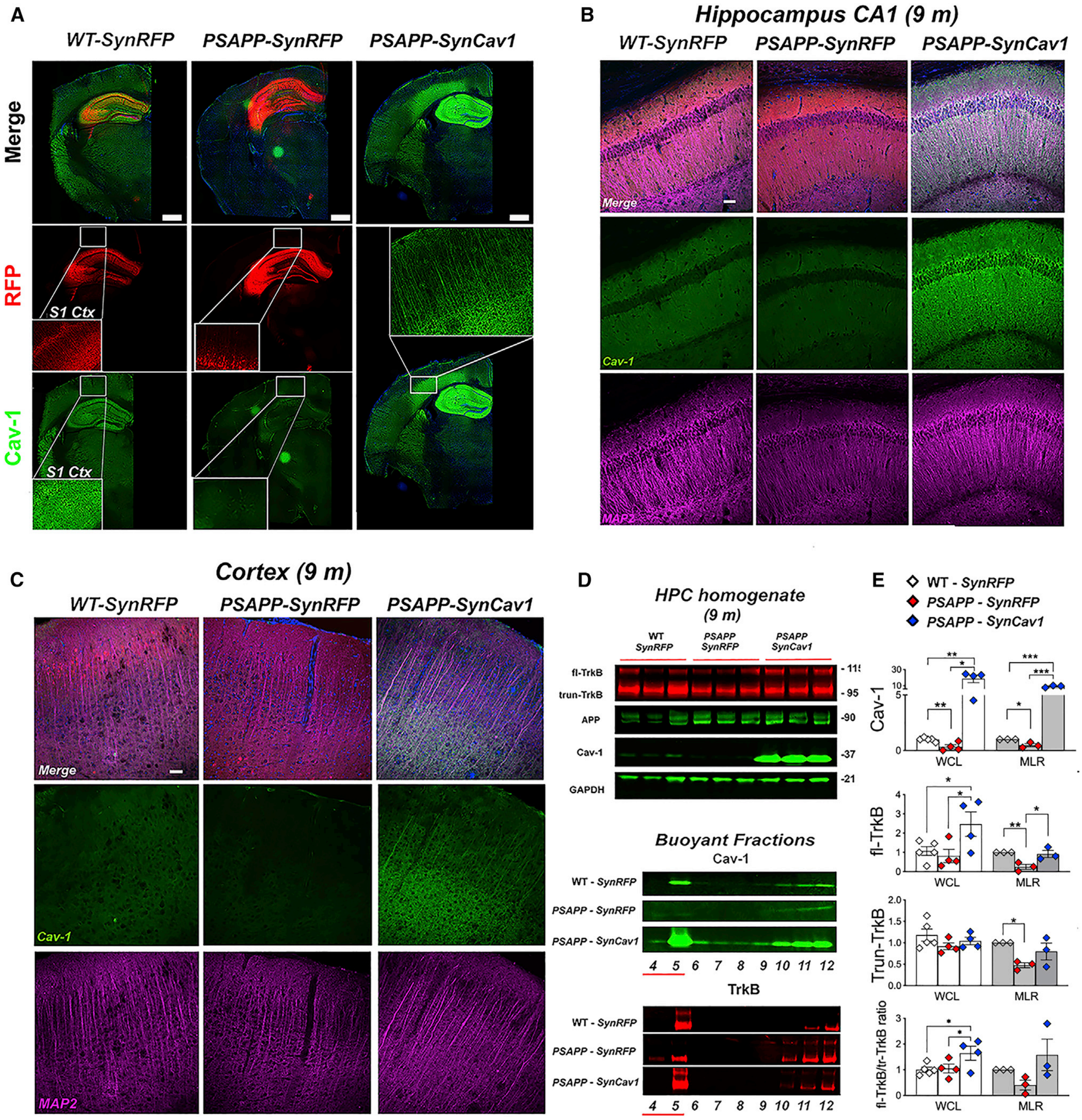


**Figure 2. *SynCav1* gene delivery preserves hippocampal MLR-localized Cav-1 and fl-TrkB expression in 9-month PSAPP mice**

(A) Immunofluorescence microscopy revealed significantly decreased Cav-1 in 9-month PSAPP mouse hippocampus, Scale bar, 500 μm. (B and C) Co-staining for Cav-1 and the dendritic marker MAP2 showed that *SynCav1* gene delivery preserved MAP2 expression in dendritic processes in the hippocampal CA1 subfields CA and cortex in 9-month PSAPP mice. Scale bar, 50 μm. IB (D and E) of 9-month hippocampal homogenates (whole cell lysate, WCL) and buoyant fractions (membrane/lipid rafts, i.e., MLRs) revealed decreased Cav-1 and full-length TrkB (fl-TrkB) expression in PSAPP-*SynRFP* mice versus WT-*SynRFP* mice. *SynCav1* preserved hippocampal MLR localized fl-TrkB expression in PSAPP mice. Fractions were generated from equal protein (0.5 μg/μL). Data present as mean ± SEM, n = 3–4 animals per group. Data were analyzed using one-way ANOVA. Significance was assumed when $p < 0.05$. *$p < 0.05$, **$p < 0.01$, ***$p < 0.001$.

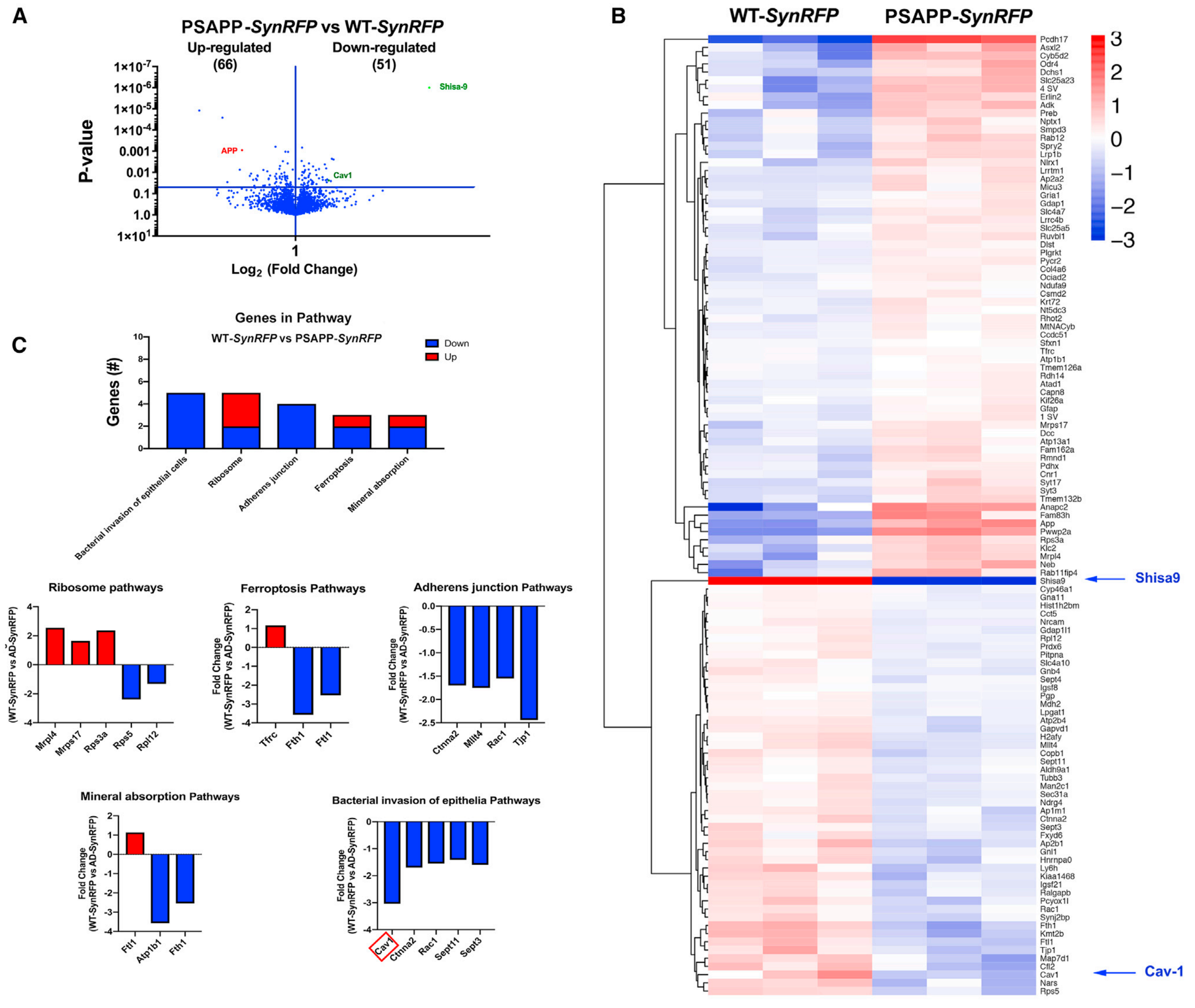


**Figure 3. Proteomics reveals altered MLR-associated proteins in PSAPP-*SynRFP* mice at 9 months**

(A) Volcano plot of 9-month MLRs between WT-*SynRFP* and PSAPP-*SynRFP* identified 117 proteins that were differentially expressed (65 upregulated and 52 downregulated). (B) Mean expression differences of significantly regulated proteins were evaluated by Euclidean hierarchical clustering. (C) Gene Ontology (GO) functional enrichment using STRING-db. Clustering revealed that Cav-1 was among the significantly downregulated in MLRs from PSAPP-*SynRFP* hippocampi. All significantly regulated quantifiable protein groups together evaluated for significant GO functional enrichment using STRING-db (https://www.string-db.org). The most significant GO terms identified were Bacterial Invasion of Epithelial Cells, Ribosome, Ferroptosis, Mineral Absorption, and Adherens Junction. All proteins in this GO term and Adherens Junction were downregulated in PSAPP-*SynRFP* versus WT-*SynRFP*. The significantly different expression levels between comparison groups were determined by two-tailed Student t test, n = 3 animals per group. Significance was assumed when *$p < 0.05$.

At 11 months, increased Cav-1 protein expression in PSAPP-*SynCav1* was sustained, with no significant difference observed between PSAPP-*SynRFP* and WT-*SynRFP* (Figures S4A and S4B). We also detected co-localization between Cav-1 and 6E10-positive plaques (Figure S4C) in 11-month PSAPP hippocampal tissue, a finding that may explain for the upregulated Cav-1 level in some 11-month PSAPP mice. While a consistent downregulation of Cav-1 was observed in 9-month PSAPP mice hippocampal tissue, no significant Cav-1 difference was measured in 11-month PSAPP due high variation among PSAPP mice.

### Proteomics analysis of MLRs reveals decreased expression of neurodegenerative proteins in PSAPP-*SynCav1* in mice

Tandem mass spectrometry (MS/MS) assessed proteins in MLRs from WT-*SynRFP*, PSAPP-*SynRFP*, and PSAPP-*SynCav1*. Quantifiable protein groups were first evaluated for significant expression

differences between WT-*SynRFP* and PSAPP-*SynRFP* (Figure 3). Of 2,417 quantifiable proteins, 66 were significantly upregulated, with mean $\log_2$ (PSAPP-*SynRFP*/WT-*SynRFP*) > 0, and 51 significantly downregulated, with mean $\log_2$ (PSAPP-*SynRFP*/WT-*SynRFP*) < 0 (Figure 3A). Mean expression differences of significantly regulated proteins were evaluated by complete Euclidean hierarchical clustering to identify protein groups of mild, moderate, or strong up- or down-regulation (Figure 3B). Cav-1 was also significantly downregulated in MLRs from PSAPP-*SynRFP* hippocampi (denoted by the red box in bacterial invasion of epithelial pathways graph), which is consistent with the IB results shown in Figure 2C. Clustering revealed Shisa9 (an AMPAR regulatory protein) was the most strongly downregulated protein in PSAPP-*SynRFP* compared to WT-*SynRFP* ($\log_2$ = −5.9). All significantly regulated quantifiable protein groups together evaluated for significant Gene Ontology (GO) functional enrichment using STRING-db (https://www.string-db.org/; Figure 3C). The most significant GO terms identified were bacterial invasion of epithelial cells, ribosome, ferroptosis, mineral absorption, and adherens junction. GO genes for the bacterial invasion of epithelia contained Cav-1, which was significantly downregulated in PSAPP-*SynRFP*.

Next, we compared PSAPP-*SynRFP* versus PSAPP-*SynCav1* (Figure 4). Of 2,417 proteins, 168 were significantly altered, with 80 significantly upregulated and 88 significantly downregulated (Figures 4A and 4B). Cav-1 was the most upregulated protein ($\log_2$ = 7.8) due to its overexpression with *SynCav1*. Shisa9 was the second most upregulated protein ($\log_2$ = 5.9), as confirmed by IB (Figure S5). Notably, several downregulated proteins in MLRs from PSAPP-*SynRFP* (ap2b1, cofilin-2, cct-5, FXYD, Kiaa1468, MAN2C1, NDRG4, synaptojanin2, Prdx6) were significantly upregulated in MLRs from PSAPP-*SynCav1*. Furthermore, several genes implicated in neurodegenerative disease pathways (e.g., AD, Parkinson's, Huntington's) were significantly downregulated in MLRs from PSAPP-*SynCav1* when compared to PSAPP-*SynRFP* (Figure 4C, graphs in blue rectangle inset). Bacterial invasion of the epithelia graph shows a significant increase in Cav-1 (Figure 4C, red rectangle). These results show that *SynCav1* decreases MLR expression of genes implicated in neurodegenerative pathways.

## *SynCav1* preserves dendritic arborization and dendritic spine number in CA1 hippocampal neurons in 11-month PSAPP mice

Hippocampal dendritic arborizations are necessary for memory formation and maintenance. Golgi-Cox staining (Figure 5A) revealed a significant reduction in CA1 apical and basal dendritic arborization, apical dendritic soma to tip distance, and dendritic spines in PSAPP-*SynRFP* mice compared to WT-*SynRFP*. PSAPP-*SynCav1* mice showed preserved apical dendritic arborization from 70 to 280 μm distance from the soma (Figure 5C), preserved CA1 basal dendritic arborization (Figure 5D), greater apical dendrite soma to tip distance (Figure 5E), and increased number of CA1 dendritic spines (Figure 5F). These findings indicate that *SynCav1* preserves structural neuroplasticity in the hippocampus of PSAPP mice that may in part explain the preserved hippocampal-dependent memory observed in Figure 1F.

## *SynCav1* preserves infrapyramidal mossy fiber area and CA3 Schaffer axon myelination in the hippocampus of PSAPP mice

Proper hippocampal circuitry (Figure 6A) is essential for cognitive function. The mossy fiber pathway, which consists of unmyelinated axons projecting from the dentate gyrus to CA3 pyramidal cells, strongly correlates with spatial learning.[15] Because PSAPP-*SynRFP* mice showed a significant reduction in hippocampal-dependent memory recall at 11 months, we measured infrapyramidal mossy fiber (IPM) and suprapyramidal mossy fiber (SPM) area using synaptoporin staining (Figure 6B).[15] As shown in Figure 6B, PSAPP-*SynRFP* mice exhibited significantly reduced IPM area compared to WT-*SynRFP*, while the PSAPP-*SynCav1* group showed significantly greater IPM area compared to PSAPP-*SynRFP*, with no significant difference in SPM area detected (Figures 6C–6E).

We further measured changes in myelin content of CA3 Shaffer collateral axons (Figures 6F and 6G), the axons projecting from CA3 pyramidal cells to CA1. Using G-ratio analysis, an inverse indicator of myelin content of an axon fiber[16] (Figure 6H), we found a significant increase in the G-ratio of PSAPP-*SynRFP* mice compared to WT-*SynRFP* at both 9 and 11 months, while PSAPP-*SynCav1* mice exhibited a significant decrease in G-ratio versus PSAPP-*SynRFP*. Further analysis revealed that the G-ratio change was due to altered myelin sheath thickness (Figure 6J) rather than axon lumen diameter (Figure 6I).

## *SynCav1* preserves ultrastructural indicators of synaptic plasticity in PSAPP mice

Loss of synapses in AD patients is closely linked to cognitive deficits and dementia.[17–20] Next, we assessed the synaptic ultrastructure of CA1 distal apical dendrites in the *stratum radiatum* using EM. Hippocampal CA1 distal apical dendrites (Figure 7A) exhibited decreased total type I excitatory asymmetric synapses (Figure 7C) and the total number of (presynaptic vesicles) PSVs/bouton (Figure 7D) in PSAPP-*SynRFP* mice versus WT-*SynRFP*, findings consistent with Scheff et al.,[18] which demonstrated decreased synapses in the *stratum radiatum* within the CA1 subfield in AD patients. When compared to PSAPP-*SynRFP*, PSAPP-*SynCav1* mice exhibited preserved total synapses and PSVs/bouton (Figures 7C and 7D). PSAPP-*SynCav1* exhibited more total PSVs/bouton versus WT-*SynRFP* at 9 months. As shown in Figures 7E–7G, we also observed altered dendritic spine morphology (i.e., more-stubby and less mushroom-like spines) in PSAPP-*SynRFP* mice compared to WT-*SynRFP*, a finding similar to that observed in human AD biopsies and AD Tg models.[21] In contrast, PSAPP-*SynCav1* mice showed preserved neck diameter (Figure 7F) and increased dendritic spine length (Figure 7G) compared to PSAPP-*SynRFP* mice. IB of MLR fractions revealed a significant decrease in PSD95 in 11 months PSAPP-*SynRFP* when compared to WT-*SynRFP*; there was no significant difference in MLR-localized PSD95 at 11 months between PSAPP-*SynCav1* and WT-*SynRFP* (Figure S6).

We next performed electrophysiology to see whether there were any alterations in long-term potentiation (LTP) based upon the microscopic anatomical and synaptic ultrastructural changes in the hippocampus. There were no changes in LTP between WT-*SynRFP* versus

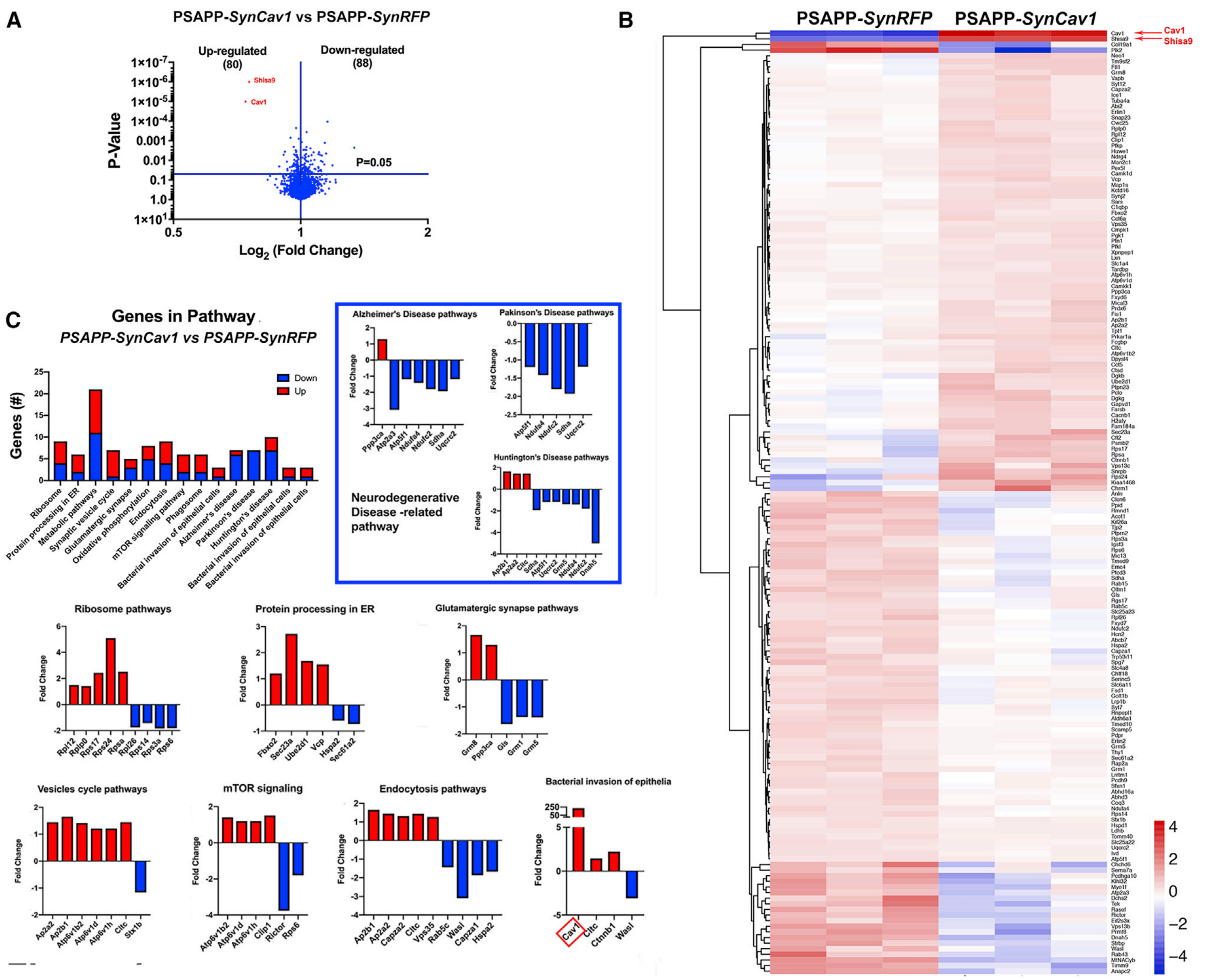


**Figure 4. Proteomics reveals that *SynCav1* decreases expression of neurodegenerative proteins in MLR fractions from 9-month PSAPP mice**

(A) Volcano plot of 9-month MLRs between PSAPP-*SynRFP* and PSAPP-*SynCav1* identified 168 proteins that were differentially expressed (80 upregulated and 88 downregulated). (B) Mean expression differences of significantly regulated proteins were evaluated by Euclidean hierarchical clustering. Cav-1 was the most upregulated protein ($log_2$ = 7.8) due to its overexpression with *SynCav1*. Notably, several downregulated proteins in MLRs from PSAPP-*SynRFP* (shisa9, ap2b1, cofilin-2, cct-5, FXYD, Kiaa1468, MAN2C1, NDRG4, synaptojanin2, Prdx6) were significantly upregulated in MLRs from PSAPP-*SynCav1*. (C) GO functional enrichment using STRING-db demonstrated that several proteins implicated in neurodegenerative disease pathways (e.g., AD, Parkinson's, Huntington's) were significantly downregulated in MLRs from PSAPP-*SynCav1* when compared to PSAPP-*SynRFP*. Red rectangular box in bacterial invasion of epithelia pathways in (C) denotes increased Cav-1. The significantly different expression levels between comparison groups were determined by two-tailed Student t test, n = 3 animals per group. Significance was assumed when $^*p < 0.05$.

PSAPP-*SynRFP* or WT-*SynRFP* versus PSAPP-*SynCav1* (Figure S7A). We additionally performed long-term depression (LTD) and found that short-term depression (1–5 min prior to LTP) was more pronounced in WT-*SynRFP* versus PSAPP-*SynRFP* with no difference in PSAPP-*SynRFP* versus either group (Figure S7B).

### *SynCav1* does not mitigate Aβ peptides (Aβ42, Aβ40, Aβ38), amyloid plaque deposition, or astrogliosis in 11-month PSAPP mice

To assess whether *SynCav1* affected amyloid plaque deposition and astrogliosis in PSAPP mice, we performed IF on brain tissue at 11 months using antibodies to 6E10. IF showed similar levels of hippocampal 6E10-positive amyloid plaques deposition in PSAPP-*SynRFP* and PSAPP-*SynCav1* mice (Figures 8A and 8B). In addition, we also measured Aβ peptides 42, 40, and 38 and total Aβ levels in hippocampus homogenates. As shown in Figure 8C, PSAPP-*SynCav1* mice showed a significant increase of brain Aβ42, Aβ40, Aβ38 and Aβ Total levels.

To assess whether *SynCav1* affected astrogliosis in PSAPP mice, we performed IF on brain tissue at 11 months using antibodies to GFAP. Both PSAPP-*SynRFP* and PSAPP-*SynCav1* brains also

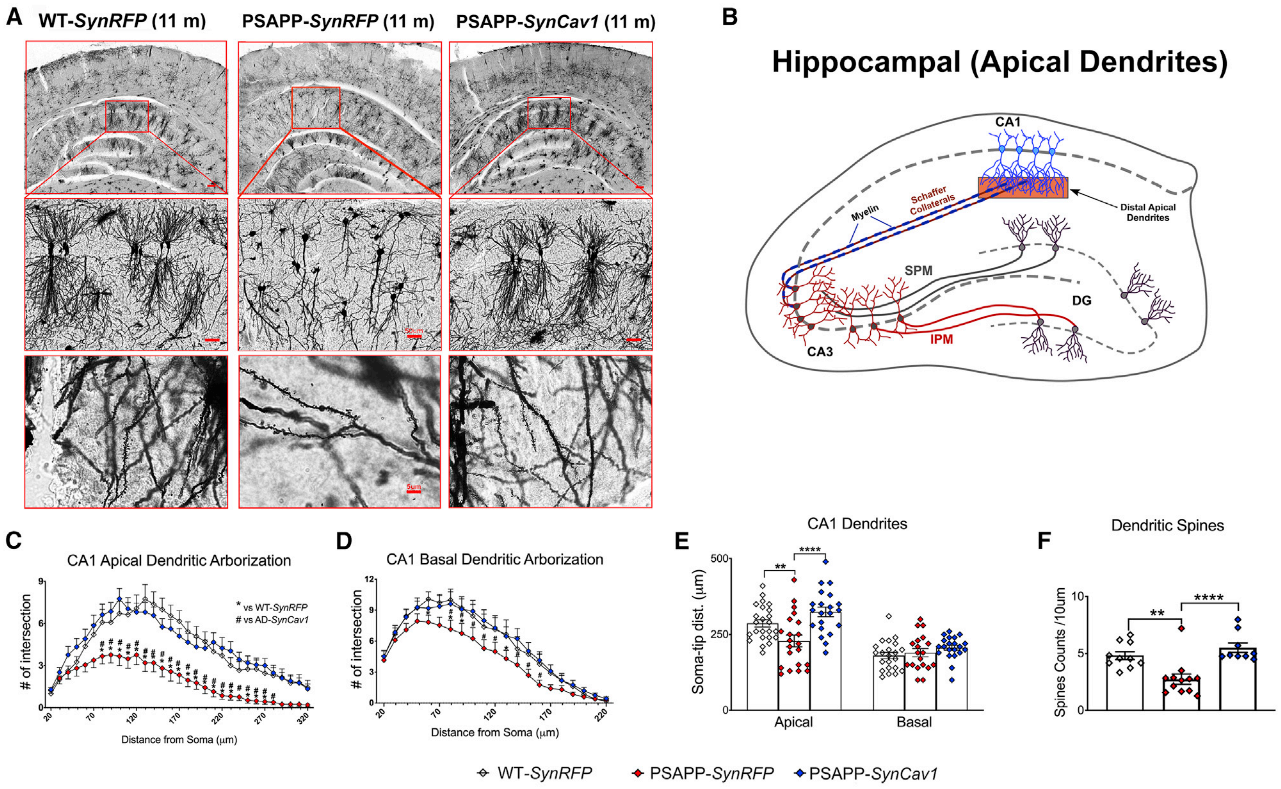


**Figure 5. *SynCav1* gene delivery preserves dendritic arborization in CA1 hippocampal neurons from 11-month PSAPP mice**

(A and B) Golgi-Cox images of *cornu ammonis* (CA1) pyramidal neurons (Top row scale bar, 200 μm; middle row images scale bar, 50 μm) (A) and schematic illustration of hippocampus with region analyzed (translucent orange rectangle) (B). Quantification revealed a significant reduction in CA1 apical and basal dendritic arborization in PSAPP-*SynRFP* mice compared to WT-*SynRFP* and PSAPP-*SynCav1* mice. (C) AD-*SynCav1* showed preserved apical dendritic arborization from 70 to 280 μm distance from the soma and basal dendritic arborization from 80 to 150 μm distance. (D) PSAPP-*SynRFP* mice also showed decreased apical dendrite soma to tip distance, which was preserved by *SynCav1*. (E) No difference in basal dendrite soma to tip distance. (F) Further analysis revealed decreased CA1 spine number in PSAPP-*SynRFP* mice and preserved dendritic spines in PSAPP-*SynCav1*. Data are presented as mean ± SEM, n = 3 animals in each group consisting of 8–12 neurons from each animal. Data were analyzed using one-way ANOVA. Significance was assumed when $p < 0.05$. *$p < 0.05$, **$p < 0.01$, ***$p < 0.001$.

exhibited elevated GFAP expression (Figures 8D and 8E), with PSAPP-*SynCav1* exhibiting a slight increase in GFAP expression compared to PSAPP-*SynRFP*. These results demonstrate that *SynCav1* did not mitigate Aβ peptides, amyloid plaque deposition, or astrogliosis in 11-month-old PSAPP mice.

## DISCUSSION

The present study is the first to demonstrate that a one-time *SynCav1* gene delivery preserves the hippocampal structure and hippocampal-dependent cognitive function in a PSAPP Tg mouse model of AD. Furthermore, *SynCav1* preserved MLR-localization of protein markers of neuronal and synaptic plasticity, all of which occurred independent of removing neurotoxic Ab plaques or reducing astrogliosis. This study builds upon previous work demonstrating the neuroprotective effects of *SynCav1* gene therapy in the setting of aging, TBI, and ALS,[8,9,14] and now further extends the therapeutic potential of *SynCav1* in another neurodegenerative disease indication, i.e., the PSAPP Tg model of AD.

Individuals with severe AD exhibit decreased neurotrophic signaling in the cortex and hippocampus,[22] thus delivery of neurotrophins[23] or increasing neurotrophin receptor (NTR) signaling could potentially reverse cognitive deficits in AD.[24,25] However, much evidence shows that functional neurotrophin signaling is dependent upon intact MLRs, Cav-1, and NTR localization to MLRs.[7,8] Pre-clinical models demonstrated that toxic Aβ or abnormal tau exposure decreased Cav-1 protein expression in neurons,[10,26] while interventions that restored or augmented Cav-1 mitigated abnormal tau accumulation, Aβ production, and reversed neurotoxicity,[10,26–28] suggesting that Cav-1 restoration may afford neuronal resilience against toxic Aβ and abnormal tau. The present study extends the knowledge that augmenting Cav-1 specifically in neurons increases fl-TrkB expression in MLRs that can undergo autophosphorylation upon agonism to activate intracellular signaling pathways necessary for neurite outgrowth, dendritic spine morphology, and cognitive performance.[29,30] Xia et al.[31] found that TrkB cleavage by delta-secreatase abolishes its phosphorylation of APP, thus enhancing AD pathology. Conversely, overexpression of normal TrkB in the hippocampus of PSAPP mice

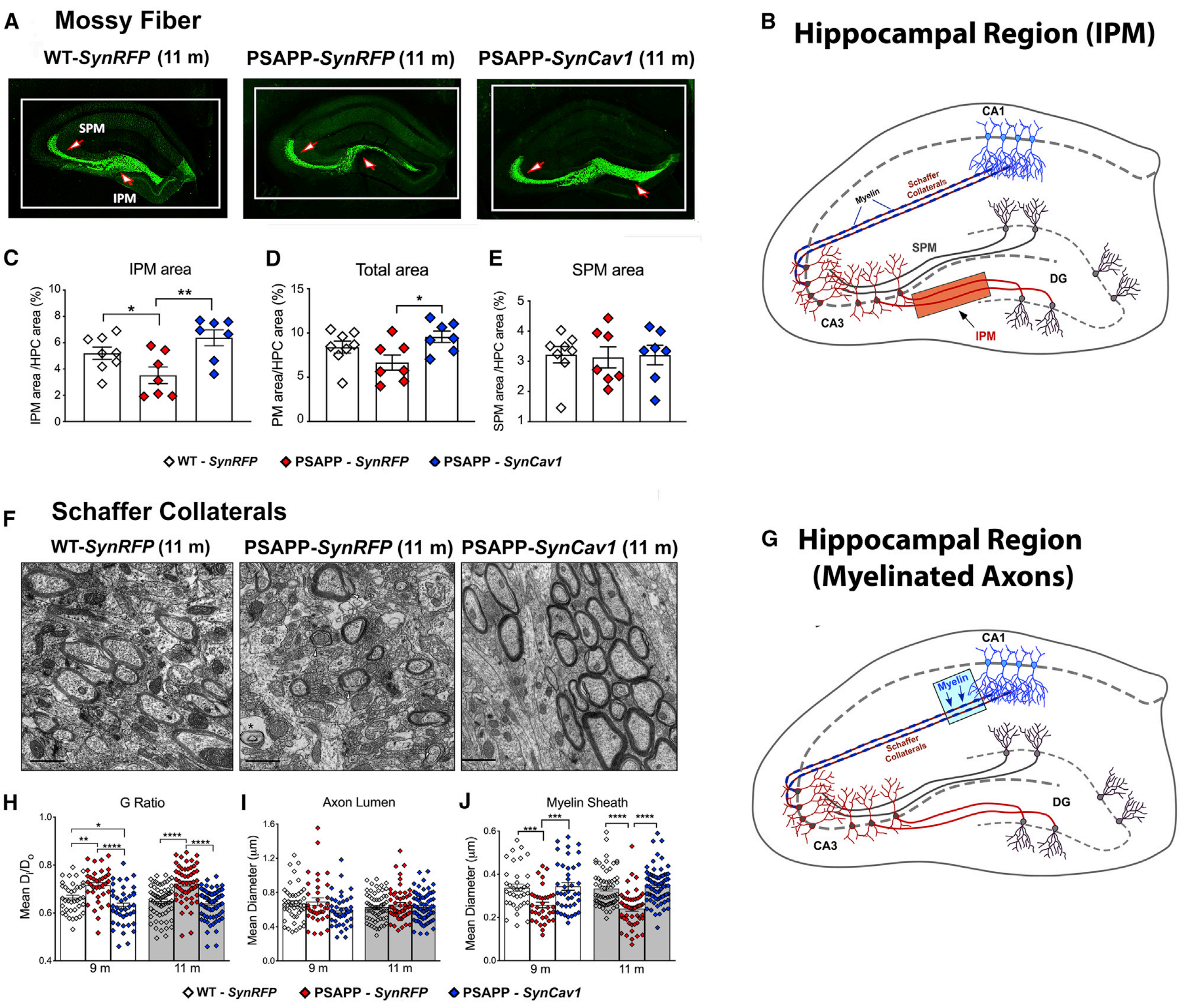


**Figure 6. *SynCav1* gene delivery preserves axonal structure and myelin content in the hippocampus of 9-month and 11-month PSAPP mice**

(A) Infrapyramidal and suprapyramidal mossy fiber (IPM and SPM) structures at 11-month (n = 3–4 animals per group consisting of 2–3 images per animal). (B) Illustration of hippocampal pathways pointing to region analyzed (translucent orange rectangle). (C–E) PSAPP-*SynRFP* mice exhibited significantly reduced IPM area (C and D) compared to WT-*SynRFP*, while the PSAPP-*SynCav1* group showed significantly greater IPM area compared to PSAPP-*SynRFP*, with no significant difference in SPM area detected (E). (F) EM images of myelinated CA3 Schaffer collateral axons. (G) Illustration of hippocampal pathways pointing to region analyzed (translucent blue rectangle). (H–J) G-ratio analysis of CA3 Schaffer collateral axons (axon lumen diameter [$D_i$]/fiber [axon lumen + myelin] diameter [$D_o$]) at 9 months and 11 months (H) revealed that the G-ratio was increased in PSAPP-*SynRFP* mice versus WT-*SynRFP*, an alteration not caused by increased axon lumen diameter (I), but rather a result of reduced myelin sheath diameter (J). PSAPP-*SynCav1* mice exhibited significantly decreased G-ratio compared to PSAPP-*SynRFP* due to increased myelin sheath diameter. Micrographs were captured at 11,000× Magnification, scale bar, 500 nm. Data are presented as mean ± SEM, n = 3–6 animals per group consisting of 10 electron micrographs images per animal. Data were analyzed using one-way ANOVA. Significance was assumed when $p < 0.05$. *$p < 0.05$, **$p < 0.01$, ***$p < 0.001$, ****$p < 0.0001$.

reduced amyloidogenic cleavage and improved cognitive function. Considering that we did not observe any significant difference in fl-TrkB expression in hippocampal homogenates in PSAPP mice, the decreased fl-TrkB on MLRs in symptomatic PSAPP mice suggests disrupted subcellular localization of fl-TrkB attributed to either disrupted MLRs or decreased Cav-1 expression within MLRs. Others have detected decreased Cav-1 transcript in degenerating neurons in postmortem human brains,[12] and a recent study out of the University of Sheffield revealed reduced Cav-1 expression and disrupted MLR in human samples as an ALS-associated risk variant.[32] Proteomics also did not detect any difference in the total TrkB, which is similar to our IB results. We also did not find a significant difference in total TrkB expression since the highly expressed Trun-TrkB dilutes any detectable change in fl-TrkB (Trun-TrkB is abundantly expressed

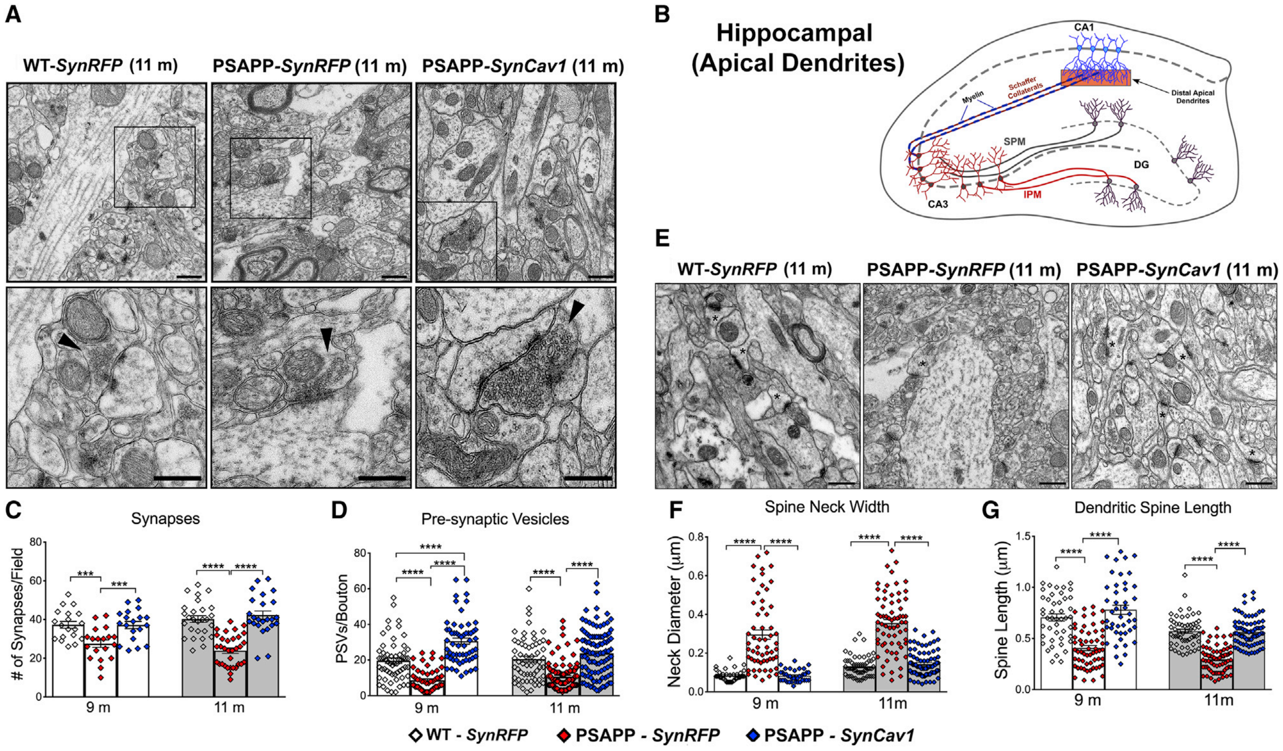


**Figure 7. *SynCav1* gene delivery preserves synaptic ultrastructure and dendritic spine morphology in the *stratum radiatum* of hippocampus from 9-month and 11-month PSAPP mice**

(A and B) EM images (A) and schematic illustration (B) of the region (translucent orange rectangle). (C and D) Quantitation of total type I asymmetric synapses (C) and presynaptic vesicles (PSVs)/axonal bouton (D) in hippocampal CA1 distal apical dendrites in the *stratum radiatum*. PSAPP-*SynRFP* mice demonstrated a significant reduction in total type I excitatory asymmetric synapses and total number of PSVs/bouton. In contrast, PSAPP-*SynCav1* mice showed a significant increase in both total synapses and total number of PSVs/bouton when compared to PSAPP-*SynRFP* and WT-*SynRFP*. Top micrographs represent 4,800× magnification, scale bar, 1 μm; bottom micrographs represent 11,000× magnification, scale bar, 500 nm. Black arrow heads denote asymmetric type I synapses. EM images (E) and quantitation of dendritic spine neck width (F) and length (G) showed that PSAPP-*SynRFP* mice exhibited altered dendritic spine morphology (i.e., more-stubby and less mushroom-like spines) with significantly increased neck diameter and reduced spine length compared to WT-*SynRFP*. PSAPP-*SynCav1* mice showed preserved neck diameter and increased spine length when compared to PSAPP-*SynRFP*. Asterisks demark the spines in the EM images (E). Micrographs represents 11,000× magnification, scale bar, 500 nm. Data are presented as mean ± SEM, n = 3–6 animals per group consisting of 10 electron micrographs per animal. Data were analyzed using one-way ANOVA within each time point. Significance was assumed when $p < 0.05$. *$p < 0.05$, **$p < 0.01$, ***$p < 0.001$, ****$p < 0.0001$.

and serves as a dominant-negative receptor). Whether the decreased MLR-localized TrkB in symptomatic PSAPP mice is due to disease or age-related altered biophysical properties of MLR-associated lipids and/or proteins[33] or due to deficits in cellular (i.e., endosomal) trafficking[34–37] is worthy of further investigation.

We have also investigated p-TrkB and p-Src in the hippocampi homogenates and MLRs. However, we observed no increase in p-TrkB or p-Src expression (Figure S3). We postulate that this variable and inconsistent p-TrkB expression to be based upon the following reasons: (1) increasing TrkB expression alone does not necessarily facilitate activation of TrkB (p-TrkB) without applying a TrkB agonist (e.g., BDNF or 7,8-dihydroxyflavone [DHF]), and (2) the kinetics of TrkB activation and subsequent downstream signaling such as p-Src, even in the presence of an applied TrkB agonist, are short in duration (15–60 min) thus making it difficult to capture this transient signaling event *in vivo* 6 months after *SynCav1* gene delivery. The preservation of MLR-associated fl-TrkB expression in PSAPP-*SynCav1* mice observed in current study may potentially increase the efficacy of endogenous BDNF or exogenous TrkB agonists (e.g., 7,8-DHF[24,38,39]).

AD brains exhibit anatomical and ultrastructural changes (i.e., reduced synapses) in hippocampal and cortical brain regions.[40] Although deposition of amyloid plaques is the traditional hallmark of AD, decreased synapses, “mushroom” spines (i.e., synaptopathy), and neuronal atrophy more closely correlate with cognitive impairment.[21,41,42] We have previously demonstrated that *SynCav1* enhanced hippocampal neuroplasticity and improved cognitive function in adult and aged WT mice.[8,16] Furthermore, *SynCav1* preserved synaptic ultrastructure, dendritic spine morphology, and axonal fiber myelin content in symptomatic PSAPP mice. The present study

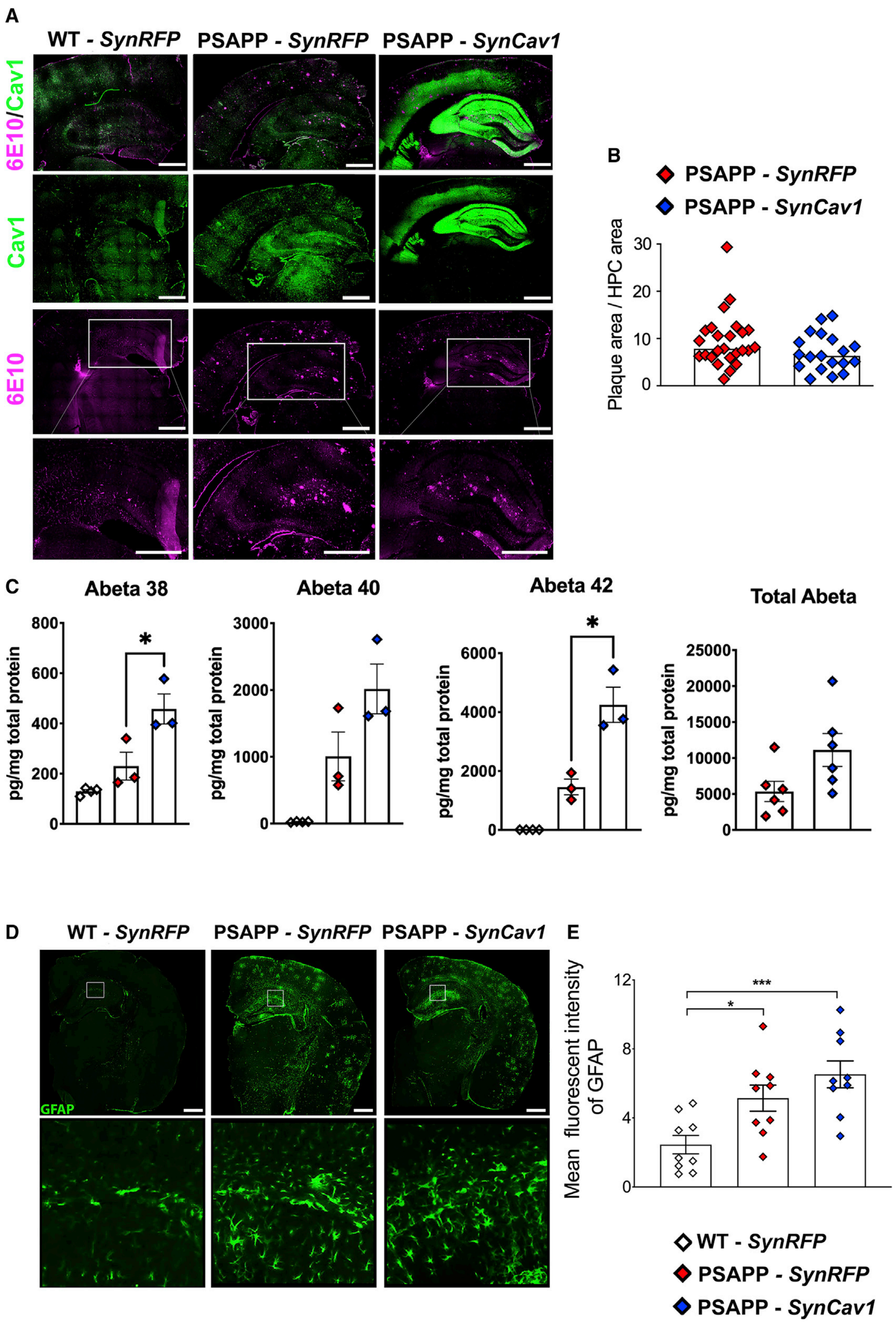


*(legend on next page)*

further extends the neuroprotective capacity of *SynCav1* gene therapy in a neurodegenerative PSAPP mouse model of AD, independent of attenuating toxic amyloid deposition or astrogliosis. Although there was no measurable difference in amyloid plaque deposition between the PSAPP mice, we did find a measurable increase in Aβ42, Aβ40, and Aβ38 in hippocampal homogenates in PSAPP-*SynCav1* mice when compared to PSAPP-*SynRFP*. These elevated Aβ levels may be caused by increased synaptic activity related to augmented synaptic vesicle trafficking and exocytosis as previously suggested[43] and could explain the increased PSVs measured by EM (Figures 7A and 7D). Interestingly, the *SynCav1*-mediated increase in Aβ38 peptide, known to be a negative regulator of Aβ42 by preventing the conversion to β sheet-rich aggregates,[44] could explain why the increase in Aβ42 did not result in an increase in amyloid plaques in PSAPP-*SynCav1* brains. Noticeable, although PSAPP-*SynRFP* mice exhibit fewer synapses at 9 months, increased PSD length and area were observed (data not shown), a subtle compensatory ultrastructural change consistent with Scheff et al. and others,[45,46] which may also explain the normal contextual memory recall observed in 11-month PSAPP mice.

The present study used a Cav-1 expression vector that is neuron-specific and does not target Cav-1 overexpression in glia cells (astrocytes or microglia). Others have shown that overexpression of MLR proteins such as Cav-1 and flotillin (FLOT) increases beta-site amyloid precursor protein cleaving enzyme (BACE) localization to MLRs and influences BACE activity[47] and increases APP processing by BACE in senescent neurons.[48] Understanding the physiological role of Cav-1 overexpression in glia cells (and microglia) versus neurons and how this influences Aβ clearance[49] or endocytosis[50] needs further investigation. Furthermore, a dose escalation study to better understand the effect varying levels Cav-1 expression has on APP processing by β- and γ-secretases could further our understanding of how Cav-1 influences APP biology.

MLRs are specialized plasmalemmal microdomains that compartmentalize synaptic signaling and adaptor proteins essential for synaptogenesis, synaptic plasticity, and cognitive function.[8,16,33] Proteomics on MLR fractions revealed a novel association between hippocampal MLRs and the AMPAR adaptor and regulatory protein Shisa9.[51–53] Shisa9 is known to regulate synaptic plasticity and memory formation in the dentate gyrus, a subregion of hippocampus.[51,52] Proteomics revealed that MLR-associated Shisa9 was significantly decreased in PSAPP mice, yet preserved with *SynCav1*, suggesting that Shisa9 may contribute to the neuroanatomical preservation of mossy fiber projections from the dentate gyrus to CA3 hippocampal subfield PSAPP-*SynCav1* mice. Additional data using co-immunoprecipitation revealed a novel interaction between Cav-1 and Shisa9 in MLR fractions (Figure S5A). We are currently researching whether *SynCav1*'s neuroprotective effect in the present study is dependent upon Shisa9/AMPAR signaling in MLRs. Additional proteomic analyses revealed reduced expression of several neurodegenerative-related pathways in MLRs from PSAPP-*SynCav1* mice, suggesting the importance of plasma membrane microdomains in modulating protein complexes involved in neuronal function and resilience against neurodegenerative conditions.

There are conflicting results regarding Cav-1 expression levels in the AD brain; while the present study and others have shown decreased Cav-1 protein and mRNA in the AD and neurodegenerative brain,[12] Gaudreault et al.[54] detected increased Cav-1 protein and mRNA in the hippocampus and frontal cortex of AD brains. It is well known that astrocytes, microglia, and vascular epithelia cells all express Cav proteins.[55–57] Thus, the elevated Cav-1 observed by Gaudreault et al.[58] may be due to prominent astrogliosis and microgliosis in AD brains. The present study observed a slight increase in Cav-1 expression in some of 11-month PSAPP mice (no significant difference with WT), and IF showed a strong co-localization between Cav-1 and 6E10-positive plaques (Figure S4). Whether the source of this increased Cav-1 adjacent to amyloid plaques is microglia or other systemic inflammatory cells needs further investigation. Considering that 9-month PSAPP mice exhibited decreased hippocampal Cav-1 expression along with learning and memory deficits, it is plausible that loss of Cav-1 expression in the hippocampus may contribute to neurodegeneration.[12,59,60] While the current study provides proof-of-concept for the neuroprotective and therapeutic potential of *SynCav1* gene therapy in PSAPP mice, we are also currently testing whether *SynCav1* delivery to PSAPP mice at later stages can reverse learning and memory deficits at later time points.

We have previously demonstrated the neuroprotective properties and neuronal resilience afforded by *SynCav1* in a variety of *in vitro* and *in vivo* models ranging from ischemia to aging to traumatic injury, as well as in neurodegenerative mice harboring a monogenic link to a familial form of ALS (i.e., hSOD1$^{G93A}$).[8,9,14,61] The current study now expands upon the therapeutic potential of *SynCav1* to yet another distinct neurodegenerative mouse model expressing a monogenic link to a familial form of AD (PSAPP). The importance of these recent findings is one more example that, regardless of the cause of the neurodegenerative condition (known versus unknown etiology, injury versus genetic abnormality), the therapeutic and translational potential of *SynCav1* might be exploited in the future to treat sporadic neurodegenerative conditions or to be used in combination with already existing drugs or biologics designed to target known monogenic candidates linked to other neurodegenerative conditions (EOFAD, FALS, Parkinson's, and Huntingtin's disease). Moreover,

**Figure 8. *SynCav1* did not mitigate hippocampal amyloid plaque deposits nor astrogliosis in 11-month PSAPP mice**
(A and B) Cav1/6E10 dual staining of 11-month PSAPP mouse and hippocampi 6E10-positive amyloid deposits quantitation at 11 months, scale bar, 500 μm. (C) Aβ38, Aβ42, and Aβ40 and AβTotal of 11-month hippocampal homogenates was measured by ELISA. (D and E) GFAP (astrocytes) staining with hippocampi quantitation axt 11 months, scale bar, 500 μm. Data were analyzed using Student t test for 6E10 analysis, n = 5–6 animals per group. Data were analyzed using one-way ANOVA for GFAP analysis, n = 3–4 animals per group. Significance was assumed when *$p < 0.05$.

the ability of *SynCav1* to preserve axonal myelin content in the hippocampus of PSAPP mice indicates its potential to combat certain demyelinating diseases such as multiple sclerosis, neuromyelitis optical, Guillain-Barre syndrome, Charcot-Marie-Tooth disease, or other forms attributed to encephalomyelitis.

In summary, the present study demonstrates that *SynCav1* gene delivery delays neurodegeneration and cognitive deficits, preserves hippocampal arborization, synaptic integrity, and axonal ultrastructure in PSAPP mice. Furthermore, we show that *SynCav1* preserves MLR-localization of the synaptic components essential for neuronal and synaptic plasticity independent of attenuating toxic Aβ plaque accumulation or astrogliosis. We are currently testing the applicability of *SynCav1* delivery at later time points to further assess its ability to not only attenuate disease progression but to also reverse neurodegeneration and associated neurobehavioral deficits. These data suggest that *SynCav1* might serve as a novel gene therapy to preserve or delay neurodegenerative conditions in AD and other forms of CNS disease of unknown etiology.

## MATERIALS AND METHODS

### Animals

PSAPP-Tg (*APPSwePS1d9*, Jackson Laboratory, number 34832)[62] and C57BL/6 mice were purchased from Jackson Laboratory (Bar Harbor, ME, USA) and bred in-house. WT littermates were used as control. All animal protocols were approved by the Veterans Administration San Diego Healthcare System Institutional Animal Care and Use Committee (#A20-030). Mice were reared (3–5/cage) with free access to food and water. AAV9-*SynRFP* was used as the control vector, and neuronal targeted overexpression of Cav-1 was achieved by AAV9-*SynCav1* viral vector (Figure 1A). At 3 months of age, WT and PSAPP mice were allocated to 3 groups randomly: WT-*SynRFP*, PSAPP-*SynRFP*, and PSAPP-*SynCav1* to receive hippocampal stereotactic injections. Brain tissue was processed for biochemistry, histopathology, immunofluorescence, and electron microscopy (EM) after behavior tests (Figure 1C).

### Genotyping

PSAPP (*APPSwePS1d9*) mice were confirmed by genomic DNA extraction and PCR using the QIAGEN DNeasy Blood and Tissue Kit (69504; QIAGEN, Valencia, CA, USA). PCR was performed for *APP* genes by using the following protocol: denaturation at 94°C for 2 min, followed by 10 cycles at 94°C for 20 s, 65°C for 15 s (−0.5 per cycle decrease), and 68°C for 10 s, then followed by 28 cycles at 94°C for 15 s, 60°C for 15 s, 72°C for 10 s, then 72°C for 2 min and hold at 10°C. All primers were purchased from Integrated DNA Technologies (Coralville, IA, USA). Primer sequences to detected Tg-positive (400 bp and 750 bp): Prp-Sense-J (mouse prion protein variant 1–2 mRNA) 5′-GGG ACT ATG TGG ACT GAT GTC GG-3′, Prp-Antisense J (mouse prion protein variant 1–2 mRNA), 5′-CCA AGC CTA GAC CAC GAG AAT GC-3′, and S36 mouse amyloid precursor protein (APP) mRNA), 5′-CCG AGA TCT CTG AAG TGA AGA TGG ATG-3′. Tg-positives were identified by two bands at 400 and 750 bp, while transgene negative presented 750 bp only. PCR products were separated on a 1% agarose gel (35 min at 135 V).

### Viral vectors and SynCav1 construct

A self-complementary AAV (scAAV) construct expressing neuron specific synapsin (Syn) promoter with the Cav-1 cDNA was generated at the UCSD Viral Vector core as previously described.[8] To link the neuron specific Syn promoter with the Cav-1 cDNA, an XbaI-SalI DNA fragment containing the Syn promoter was inserted into the NheI-SalI sites of pEGFP-N1 (Clontech). The resulting plasmid was designated pSyn-EGFP. A 537-bp Cav-1 cDNA was isolated from the pCRII-TOPO vector (Invitrogen) by digestion with PmeI-NotI and inserted into the SmaI-NotI site of the pSyn-EGFP to generate pSyn-Cav-1, in which the EGFP gene was replaced with Cav-1 cDNA.[7] The Syn-promoter-Cav-1 cassette was isolated from pSyn-Cav-1. The scAAV vector construct, expressing Cav-1 or RFP driven by the Syn promoter was made by DNA synthesis of the Syn promoter (480 bp) through Cav-1 (537 bp) or enhanced RFP (700 bp) and cloning it into an scAAV backbone plasmid. The scAAV9-*SynCav1* or scAAV9-*SynRFP* vectors were produced by transient transfection of HEK293T cells with each vector plasmid, pRep2/Cap9, and pAd-Helper plasmid. Helper virus-free AAV vectors were produced by Polyethylenimine (PEI)-mediated transient co-transfection of HEK293T cells with three plasmids (pVector, pRep2/CapX [X stands for each serotype] and pAd-Helper). Cells were collected at 72 h post-transfection and cell lysates were made by 3× freeze/thaw cycle. AAV vectors in the cell lysates were purified by combination of sucrose-cushion ultracentrifugations and anion-exchange column chromatography. Virus titers were measured by quantitative real-time PCR to determine genome copy number in the vector preparations (gc/mL) as a measure of AAV particles with full genome content.

### Stereotactic injection

3-month-old mice were mounted onto a stereotaxic frame under anesthesia (2% isoflurane). Bilateral burr holes were made by a 22-gauge needle. Hippocampal injections using 33-gauge, 10 μL Gas Tight syringe (Hamilton, Reno, NV, USA) were controlled by injectomate (Neurostar, Berlin, Germany). 1.5 μL of AAV9 (viral titer: $10^9$ genome copies [g.c.]/μL) containing synapsin-red fluorescent protein or synapsin-Cav-1 (*SynCav1*) was injected bilaterally over 180 s at three locations (1st site: AP, 1.82 mm; Lat, 1.15 mm; DV, 1.7 mm; 2nd site: AP, 2.30 mm; Lat, 2.25 mm; DV, 1.75 mm; 3rd site: AP, 2.80; Lat, 2.5 mm; DV, 2.00 mm) with 1 min indwelling time as previously described.[8] Figure 1B and Video S1 display broad brain tissue AAV infectivity and transgene expression (whole hippocampus rostral to caudal, dorsal to ventral, and portions of the somatosensory and parietal cortex) using *SynRFP*.

### Open field and fear conditioning behavior

Open field and fear conditioning were performed as previously described.[8] Locomotion was recorded for 10 min and analyzed by a computerized video tracking system (Noldus XT 7.1, Leesburg, VA, USA). Recorded parameters included distance moved (cm), velocity (cm/second), and time spent in the center of the arena (seconds).

For fear conditioning, presentation of unconditioned stimuli (US; foot-shock) and conditioned stimuli (CS; auditory tone) were controlled using Med Associates (St. Albans, VT, USA), and movement was monitored by video. Freezing protocol (Figure 1D) was determined using Video Freeze (Med Associates; San Diego Instruments, San Diego, CA, USA).[8,15]

### Biochemical characterization of MLRs

Hippocampi were homogenized at 4°C in 500 mM sodium carbonate (pH 11.0; containing protease and phosphatase inhibitors) and then sonicated 3× for 15 s. Samples (0.5 mg/mL) were subjected to sucrose density gradient fractionation as previously described.[8] Briefly, lysate was normalized to 0.5 mg/mL and mixed with equal volume of 80% sucrose in MBS (25 mM MES and 150 mM NaCl, pH 6.5) to generate 40% sucrose in MBS. 2 mL of 40% sucrose/sample was followed by layering 6 mL of 35% sucrose and 4 mL of 5% sucrose. Gradients were ultracentrifuged using a SW-41 rotor at 39k rpm for 17 h at 4°C. Fractions (1 mL) were collected from 4 to 12 mL. Samples were run as individual fractions 4–12 and subjected to IB assays. Buoyant fractions 4 and 5 (i.e., MLR fractions) are found at the 5%/35% interface based upon their lipid components and biophysical properties. Homogenates and fractions were immunoblotted using primary antibodies Cav-1 (Cell Signaling #3238; 1:1,000), TrkB (BD Biosciences 610102; 1:1,000), and GAPDH (Cell Signaling #2118s; 1:1,000) overnight at 4°C followed by incubation with IR-dye labeled secondary antibody for 1 h and measured with Li-Cor Odyssey followed by densitometric analysis.

### Proteomics

MLRs protein samples were diluted in TNE (50 mM Tris pH 8.0, 100 mM NaCl, 1 mM EDTA) buffer. RapiGest SF reagent (Waters) was added to the mix (0.1% final concentration) and samples boiled for 5 min. 1 mM TCEP (Tris [2-carboxyethyl] phosphine) was added to the samples and incubated at 37°C for 30 min. Samples were carboxymethylated with 0.5 mg/mL of iodoacetamide for 30 min at 37°C followed by neutralization with 2 mM TCEP. Samples were digested with trypsin (trypsin: protein ratio 1:50) overnight at 37°C. RapiGest was degraded and removed by treating the samples with 250 mM HCl at 37°C for 1 h followed by centrifugation at 14k rpm for 30 min at 4°C. The soluble fraction was then added to a new tube and the peptides were extracted and desalted using C18 desalting columns (Thermo Scientific, PI-87782). Peptides were quantified using BCA assay and a total of 1 μg of peptides were injected for liquid chromatography-MS (LC-MS) analysis.

### LC-MS-MS

Trypsin-digested peptides were analyzed by ultrahigh pressure LC (UPLC) coupled with tandem MS (LC-MS/MS) using nano-spray ionization using an Orbitrap fusion Lumos hybrid mass spectrometer (Thermo) interfaced with nano-scale reversed-phase UPLC (Thermo Dionex UltiMate 3000 RSLC nano System) using a 25 cm, 75-μm ID glass capillary packed with 1.7-μm C18 (130) BEHTM beads (Waters). Protein identification and label free quantification was carried out using Peaks Studio 8.5 (Bioinformatics Solutions). Proteins were considered identified if the false discovery rate (FDR) was less than 1% of peptide-spectrum matches to *m. musculus* database (Uniprot/SwissProt) compared to the decoy-fusion library.

### Bioinformatics

Quantifiable protein levels were expressed as peak area and compared as relative intensity for WT-*SynRFP* versus PSAPP-*SynRFP* or PSAPP-*SynRFP* versus PSAPP-*SynCav1* with significance set at $p < 0.05$. Mean significantly different expression levels were displayed as heatmaps for up- or downregulation using Euclidean clustering with complete linkage. Significantly up or downregulated quantifiable protein groups were subjected to GO using STRING-db (https://www.string-db.org/). The GO terms with the lowest FDR (all less than 1%) were displayed. Significantly regulated GO terms are displayed as $\log_2$ fold change of genes.

### Golgi-cox staining

Brains were submerged in Golgi-Cox solution A + B (FD Neurotechnologies, Ellicot City, MD, USA) for 8 days followed by solution C for 4 days at room temperature (RT). 80-μm-thick coronal cryosections were prepared for staining with solution D + E and dehydrated according to the manufacturer's instructions. Three mice per group were used for Golgi-Cox staining. To evaluate hippocampal neuron dendritic morphology, we used a Zeiss AxioImager microscope and a computer-based system (Neurolucida; MicroBrightField) to generate three-dimensional neuron tracings that were subsequently visualized and analyzed using NeuroExplorer (MicroBrightField). For each animal, five to eight CA1 pyramidal neurons were traced at 40× magnification with an oil immersion lens. For CA1 neurons, both the apical and basal dendrites were traced and analyzed separately as described in Mandyam et al.[8] To evaluate hippocampal neuron spine morphology, we visualized spines by 100× magnification and traced by Adobe Photoshop (San Jose, CA, USA). For each animal, three to five dendritic branches were traced and analyzed for dendritic spines.

### IF microscopy

Floating sections were blocked with 10% goat serum and incubated with chicken anti-MAP-2 (1:250, Abcam, #ab5392, USA) and rabbit anti-Cav-1 (1:1,000, Cell Signaling, #3267, USA) at 4°C overnight. Slices were then incubated with species-specific fluorescence secondary antibody in the dark for 1 h and preserved with anti-fade DAPI-mounting medium. For Aβ plaques staining, sections were incubated in 98% formic acid for 5 min to expose the epitope before the blocking process and purified Alex Fluor 488 anti-β-Amyloid 1-16 antibody (1:200, BioLegend, #39347, USA) was used to visualize amyloid plaques.

### ELISA

Hippocampal homogenates (same as the IB samples) from 9-month-old WT-*SynRFP* (n = 4), PSAPP-*SynRFP* (n = 3), and PSAPP-*SynCav1* (n = 3) were tested using the Meso Scale Discovery Aβ triplex immunoassay with 4G8 detection (catalog # K15141E-1, MSD Rockville, MD, USA) to quantitate levels of Aβ42, Aβ40, and Aβ38. The

kit was run according to the manufacturer's instructions. Briefly, 25 μL of SULFO-TAG conjugated 4G8 detection antibody and 25 μL of WT sample or 3 μL of AD samples were added to a blocked MSD multiplex plate pre-coated with capture antibodies specific for Aβ42, Aβ40, and Aβ38 and incubated for 2 h with shaking at RT. The plate was read on an MSD SECTOR Imager 2400 after addition of read buffer. Concentrations of Aβ42, Aβ40, and Aβ38 in samples were backfitted from the respective standard curves using MSD Workbench software. Total Abeta was quantified in the AD brain samples using Meso Scale Discovery's Electrochemiluminescence R-plex assay (MSD, USA). The assay was performed following the manufacturer's instruction using MSD GOLD 96-well small spot streptavidin plate and recommended diluents. Samples were tested at 1:8 dilution. Plates were analyzed on an MSD SECTOR Imager 2400 instrument. All data was normalized by protein concentration measure by Bradford assay. 3–4 mice per group was used to get quantifiable data.

### Electrophysiology

Hippocampal slices were prepared as previously described.[16] Mice were anesthetized with isoflurane before decapitation. The brain was quickly removed and immersed for 2 min in ice-cold low-calcium "modified" artificial cerebrospinal fluid (mACSF) composed of (in mM): 119 NaCl, 2.5 KCl, 1.3 $CaCl_2$, 2.7 $MgSO_4$, 1 $NaH_2PO_4$, 26 $NaHCO_3$, and 10 glucose, osmolarity ~305 mOsm, continuously bubbled with 95% $O_2$%–5% $CO_2$, pH 7.4. The dorsal hippocampus was dissected out and cut in ice-cold mACSF with a vibratome (Leica VT1000S; Nussloch) into 350 μm thick slices from the middle part of the hippocampus. The slices were allowed to recover in oxygenated mACSF at 33°C for 20 min, and then at RT for an additional 1.5–5 h before experimental recordings. Slices were transferred into the recording chamber and superfused with regular ACSF containing (in mM): 119 NaCl, 2.5 KCl, 2.5 $CaCl_2$, 1.3 $MgSO_4$, 1 $NaH_2PO_4$, 26 $NaHCO_3$, and 10 glucose, continuously bubbled with 95% $O_2$%–5% $CO_2$, pH 7.4, osmolarity ~305 mOsm, at a constant rate of 2.5 mL/min at 32°C. Recording electrodes were made of borosilicate glass capillaries (1B150F, World Precision Instruments) using a Sutter P-87 electrode puller (Sutter Instruments) and filled with ACSF (resistance ~0.8–1MΩ). Monopolar stimulating electrodes were made of Pt/Ir (Platinum/Iridium) wires of diameter 25.4 μm (PTT0110, World Precision Instruments) with 100-μm-long exposed tips. Both the stimulating and recording electrodes were inserted under visual control perpendicular to the slice surface into the CA1 "stratum radiatum" at a distance 250–300 μm from each other. The initial slope of field excitatory postsynaptic potentials (fEPSPs) was measured at latencies 0.1–0.9 ms. Testing stimuli (duration 100 μs, current 70 μA) evoked field responses with amplitudes of 70%–80% of maximum. After stabilization of responses, input–output dependences were measured using series of stimulation intensities (range 10–150 μA). LTP was induced by high-frequency tetanization (70 μA, 100 Hz for 1 s) and was recorded 70 min after tetanus.

### EM

EM was performed as previously described.[15] Briefly, mice were perfused with saline and 2% glutaraldehyde via left ventricle. The fixed brain was initially sliced into 8 separate 1 mm coronal brain sections using a mouse brain mold slicer. Coronal brain section covering −1.5 to −2.5 Bregma (as determined by the Mouse Brain Atlas) was transferred to the UCSD EM core. Brains were carefully removed and fixed with 2% glutaraldehyde, treated with 1% $OsO_4$, and were then en bloc stained with uranyl acetate. At the UCSD EM core, the brain section was visually trimmed to CA1 regions encompassing the pyramidal cell bodies (stratum pyramidale) along and the distal apical dendrites within the stratum radiatum of the hippocampus and thin sectioned to 70 nm as previously described.[15,16] For flat embedding of the sections, thin flexible molds were employed to lay the sections as level as possible in LX112 embedding media and then overlaid with plastic coverslips. Grids were viewed unstained using FEI Tecnai EM scope. Excitatory synapses were identified by the presence of a prominent, asymmetric postsynaptic density (PSD), and PSVs were normalized to the total boutons per field. Morphological dendritic spines were measured as previously described.[21] G-ratio was defined as the diameter of the axon lumen divided by the diameter of the fiber (axon lumen plus myelin).[16]

### Statistical analyses

All data presented in this study were representative data from at least two independent cohorts, except the MS data. The number of sample size are indicated under each section of the Materials and methods and in the figure legends. Before determining statistical significance, data were checked for normal distribution. For all behavior and histology, data were analyzed by one-way analysis of variance (ANOVA) or two-way ANOVA followed by Fisher's LSD or Tukey's multiple comparisons tests as appropriate, For immunoblot, t test was used to compare Cav-1 expression due to extremely high level of exogenous Cav-1 expression introduced by *SynCav1*, one-way ANOVA followed by Fisher's LSD was used to compare the quantitative of TrkB in whole-cell lysates and MLR samples separately using GraphPad Prism 6 (La Jolla, CA, USA). Data were presented as mean ± SEM and significance assumed when *$p < 0.05$. Experimental groups were blinded to the observer and code was broken for analysis.

### Availability of data and materials

The data generated and/or analyzed during this are included in the article and materials are available from the corresponding authors on reasonable request.

## SUPPLEMENTAL INFORMATION

Supplemental information can be found online at https://doi.org/10.1016/j.omtm.2021.03.021.

## ACKNOWLEDGMENTS

This manuscript is dedicated to the memory of Patrick J. Head and Marilyn G. Farquhar. Thanks to Alice Zemljic-Harpf (Anesthesiology) and Ying Jones (UCSD EM core) for the preparation of the brain samples and training on the EM and Dr. Marilyn Farquhar for consultation. Electron micrographs were taken in the Cellular and Molecular Medicine EM core facility supported in part by National Institutes of Health Award number S10OD023527. Thanks

to Robert Rissman, Director of UCSD Alzheimer's Disease Cooperative Study (ADCS) Biomarker Core and AD Research Center (ADRC) Neuropathology Core, for a technical assistant with amyloid plaque immunofluorescence. Thanks to Chitra Mandyam for consultation on behavior. Work in the authors' laboratories is supported by Veterans Affairs Merit Award from the Department of Veterans Affairs BX003671 (B.P.H.) and National Institutes of Health, Bethesda, MD, USA (NS073653 to B.P.H., GM085179 to P.M.P., and R56AG057469 and R01NS109075 to V.H.). J.Q.T. was supported by the Penn AD Core Center grant (AG-10124-28).

## AUTHOR CONTRIBUTIONS

S.W. performed experiments, data analyses, and wrote and edited the manuscript; J.S.L. performed genotyping, colony maintenance, and biochemical experiments; S.P. and V.H. assisted with proteomic analyses and bioinformatics; N.K. performed behavioral studies; P.S. performed biochemical experiments; M.D. performed biochemical experiments; K.Z. assisted with vector gene delivery; I.C.K. assisted with Golgi-Cox analyses; T.Z. performed the light sheet microscopy; A.M. generated the AAV vector constructs; P.N. assisted with Meso Scale Discovery ELISA; A.K. assisted with experimental design; S.L.W. assisted with experimental design and editing; J.Q.T. assisted with microscopic experimental design and editing; D.M.R., H.H.P., and P.M.P. assisted with editing; B.P.H. supervised overall design and concept, performed data analyses, and wrote and edited the manuscript. All authors read and approved the final manuscript.

## DECLARATION OF INTERESTS

The authors declare no competing interests.

## REFERENCES


1. Eggers, R., de Winter, F., Hoyng, S.A., Hoeben, R.C., Malessy, M.J.A., Tannemaat, M.R., and Verhaagen, J. (2019). Timed GDNF gene therapy using an immune-evasive gene switch promotes long distance axon regeneration. Brain *142*, 295–311.
2. Bouaita, A., Augustin, S., Lechauve, C., Cwerman-Thibault, H., Bénit, P., Simonutti, M., Paques, M., Rustin, P., Sahel, J.A., and Corral-Debrinski, M. (2012). Downregulation of apoptosis-inducing factor in Harlequin mice induces progressive and severe optic atrophy which is durably prevented by AAV2-AIF1 gene therapy. Brain *135*, 35–52.
3. Morgado, I., and Garvey, M. (2015). Lipids in Amyloid-β Processing, Aggregation, and Toxicity. Adv. Exp. Med. Biol. *855*, 67–94.
4. Fabelo, N., Martín, V., Marín, R., Moreno, D., Ferrer, I., and Díaz, M. (2014). Altered lipid composition in cortical lipid rafts occurs at early stages of sporadic Alzheimer's disease and facilitates APP/BACE1 interactions. Neurobiol. Aging *35*, 1801–1812.
5. Deverman, B.E., Ravina, B.M., Bankiewicz, K.S., Paul, S.M., and Sah, D.W.Y. (2018). Gene therapy for neurological disorders: progress and prospects. Nat. Rev. Drug Discov. *17*, 641–659.
6. Combs, B., Kneynsberg, A., and Kanaan, N.M. (2016). Gene Therapy Models of Alzheimer's Disease and Other Dementias. Methods Mol. Biol. *1382*, 339–366.
7. Head, B.P., Hu, Y., Finley, J.C., Saldana, M.D., Bonds, J.A., Miyanohara, A., Niesman, I.R., Ali, S.S., Murray, F., Insel, P.A., et al. (2011). Neuron-targeted caveolin-1 protein enhances signaling and promotes arborization of primary neurons. J. Biol. Chem. *286*, 33310–33321.
8. Mandyam, C.D., Schilling, J.M., Cui, W., Egawa, J., Niesman, I.R., Kellerhals, S.E., Staples, M.C., Busija, A.R., Risbrough, V.B., Posadas, E., et al. (2017). Neuron-Targeted Caveolin-1 Improves Molecular Signaling, Plasticity, and Behavior Dependent on the Hippocampus in Adult and Aged Mice. Biol. Psychiatry *81*, 101–110.
9. Egawa, J., Schilling, J.M., Cui, W., Posadas, E., Sawada, A., Alas, B., Zemljic-Harpf, A.E., Fannon-Pavlich, M.J., Mandyam, C.D., Roth, D.M., et al. (2017). Neuron-specific caveolin-1 overexpression improves motor function and preserves memory in mice subjected to brain trauma. FASEB J. *31*, 3403–3411.
10. Yuan, D., Zeng, C., Chen, Q., Wang, F., Yuan, L., Zhu, Y., Shu, Z., and Chen, N. (2017). Root-Securing and Brain-Fortifying Liquid Upregulates Caveolin-1 in Cell Model with Alzheimer's Disease through Inhibiting Tau Phosphorylation. Neurol. Res. Int. *2017*, 6248351.
11. Flis, D.J., Dzik, K., Kaczor, J.J., Halon-Golabek, M., Antosiewicz, J., Wieckowski, M.R., and Ziolkowski, W. (2018). Swim Training Modulates Skeletal Muscle Energy Metabolism, Oxidative Stress, and Mitochondrial Cholesterol Content in Amyotrophic Lateral Sclerosis Mice. Oxid. Med. Cell. Longev. *2018*, 5940748.
12. Mufson, E.J., He, B., Ginsberg, S.D., Carper, B.A., Bieler, G.S., Crawford, F., Alvarez, V.E., Huber, B.R., Stein, T.D., McKee, A.C., and Perez, S.E. (2018). Gene Profiling of Nucleus Basalis Tau Containing Neurons in Chronic Traumatic Encephalopathy: A Chronic Effects of Neurotrauma Consortium Study. J. Neurotrauma *35*, 1260–1271.
13. Tiernan, C.T., Ginsberg, S.D., He, B., Ward, S.M., Guillozet-Bongaarts, A.L., Kanaan, N.M., Mufson, E.J., and Counts, S.E. (2018). Pretangle pathology within cholinergic nucleus basalis neurons coincides with neurotrophic and neurotransmitter receptor gene dysregulation during the progression of Alzheimer's disease. Neurobiol. Dis. *117*, 125–136.
14. Sawada, A., Wang, S., Jian, M., Leem, J., Wackerbarth, J., Egawa, J., Schilling, J.M., Platoshyn, O., Zemljic-Harpf, A., Roth, D.M., et al. (2019). Neuron-targeted caveolin-1 improves neuromuscular function and extends survival in $SOD1^{G93A}$ mice. FASEB J. *33*, 7545–7554.
15. Pearn, M.L., Schilling, J.M., Jian, M., Egawa, J., Wu, C., Mandyam, C.D., Fannon-Pavlich, M.J., Nguyen, U., Bertoglio, J., Kodama, M., et al. (2018). Inhibition of RhoA reduces propofol-mediated growth cone collapse, axonal transport impairment, loss of synaptic connectivity, and behavioural deficits. Br. J. Anaesth. *120*, 745–760.
16. Egawa, J., Zemljic-Harpf, A., Mandyam, C.D., Niesman, I.R., Lysenko, L.V., Kleschevnikov, A.M., Roth, D.M., Patel, H.H., Patel, P.M., and Head, B.P. (2018). Neuron-Targeted Caveolin-1 Promotes Ultrastructural and Functional Hippocampal Synaptic Plasticity. Cereb. Cortex *28*, 3255–3266.
17. Selkoe, D.J. (1991). The molecular pathology of Alzheimer's disease. Neuron *6*, 487–498.
18. Scheff, S.W., Price, D.A., Schmitt, F.A., DeKosky, S.T., and Mufson, E.J. (2007). Synaptic alterations in CA1 in mild Alzheimer disease and mild cognitive impairment. Neurology *68*, 1501–1508.
19. Hyman, B.T., Phelps, C.H., Beach, T.G., Bigio, E.H., Cairns, N.J., Carrillo, M.C., Dickson, D.W., Duyckaerts, C., Frosch, M.P., Masliah, E., et al. (2012). National Institute on Aging-Alzheimer's Association guidelines for the neuropathologic assessment of Alzheimer's disease. Alzheimers Dement. *8*, 1–13.
20. Honig, L.S., Vellas, B., Woodward, M., Boada, M., Bullock, R., Borrie, M., Hager, K., Andreasen, N., Scarpini, E., Liu-Seifert, H., et al. (2018). Trial of Solanezumab for Mild Dementia Due to Alzheimer's Disease. N. Engl. J. Med. *378*, 321–330.
21. Androuin, A., Potier, B., Nägerl, U.V., Cattaert, D., Danglot, L., Thierry, M., Youssef, I., Triller, A., Duyckaerts, C., El Hachimi, K.H., et al. (2018). Evidence for altered dendritic spine compartmentalization in Alzheimer's disease and functional effects in a mouse model. Acta Neuropathol. *135*, 839–854.
22. Ferrer, I., Marín, C., Rey, M.J., Ribalta, T., Goutan, E., Blanco, R., Tolosa, E., and Martí, E. (1999). BDNF and full-length and truncated TrkB expression in Alzheimer disease. Implications in therapeutic strategies. J. Neuropathol. Exp. Neurol. *58*, 729–739.
23. Tuszynski, M.H., Thal, L., Pay, M., Salmon, D.P., U, H.S., Bakay, R., Patel, P., Blesch, A., Vahlsing, H.L., Ho, G., et al. (2005). A phase 1 clinical trial of nerve growth factor gene therapy for Alzheimer disease. Nat. Med. *11*, 551–555.
24. Castello, N.A., Nguyen, M.H., Tran, J.D., Cheng, D., Green, K.N., and LaFerla, F.M. (2014). 7,8-Dihydroxyflavone, a small molecule TrkB agonist, improves spatial memory and increases thin spine density in a mouse model of Alzheimer disease-like neuronal loss. PLoS ONE *9*, e91453.

25. Beeri, M.S., and Sonnen, J. (2016). Brain BDNF expression as a biomarker for cognitive reserve against Alzheimer disease progression. Neurology *86*, 702–703.

26. Wu, J., Zhou, S.L., Pi, L.H., Shi, X.J., Ma, L.R., Chen, Z., Qu, M.L., Li, X., Nie, S.D., Liao, D.F., et al. (2017). High glucose induces formation of tau hyperphosphorylation via Cav-1-mTOR pathway: A potential molecular mechanism for diabetes-induced cognitive dysfunction. Oncotarget *8*, 40843–40856.

27. Kapoor, A., Hsu, W.M., Wang, B.J., Wu, G.H., Lin, T.Y., Lee, S.J., Yen, C.T., Liang, S.M., and Liao, Y.F. (2010). Caveolin-1 regulates γ-secretase-mediated AβPP processing by modulating spatial distribution of γ-secretase in membrane. J. Alzheimers Dis. *22*, 423–442.

28. Zhao, Y.L., Song, J.N., Ma, X.D., Zhang, B.F., Li, D.D., and Pang, H.G. (2016). Rosiglitazone ameliorates diffuse axonal injury by reducing loss of tau and up-regulating caveolin-1 expression. Neural Regen. Res. *11*, 944–950.

29. Fenner, B.M. (2012). Truncated TrkB: beyond a dominant negative receptor. Cytokine Growth Factor Rev. *23*, 15–24.

30. Psotta, L., Rockahr, C., Gruss, M., Kirches, E., Braun, K., Lessmann, V., Bock, J., and Endres, T. (2015). Impact of an additional chronic BDNF reduction on learning performance in an Alzheimer mouse model. Front. Behav. Neurosci. *9*, 58.

31. Xia, Y., Wang, Z.H., Liu, P., Edgington-Mitchell, L., Liu, X., Wang, X.C., and Ye, K. (2020). TrkB receptor cleavage by delta-secretase abolishes its phosphorylation of APP, aggravating Alzheimer's disease pathologies. Mol. Psychiatry. Published online August 11, 2020. https://doi.org/10.1038/s41380-020-00863-8.

32. Cooper-Knock, J., Zhang, S., Kenna, K.P., Moll, T., Franklin, J.P., Allen, S., Nezhad, H.G., Iacoangeli, A., Yacovzada, N.Y., Eitan, C., et al.; Project MinE ALS Sequencing Consortium (2021). Rare variant burden analysis within enhancers identifies CAV1 as an ALS risk gene. Cell Rep. *34*, 108730.

33. Egawa, J., Pearn, M.L., Lemkuil, B.P., Patel, P.M., and Head, B.P. (2016). Membrane lipid rafts and neurobiology: age-related changes in membrane lipids and loss of neuronal function. J. Physiol. *594*, 4565–4579.

34. Chevalier-Larsen, E., and Holzbaur, E.L. (2006). Axonal transport and neurodegenerative disease. Biochim. Biophys. Acta *1762*, 1094–1108.

35. Poon, W.W., Blurton-Jones, M., Tu, C.H., Feinberg, L.M., Chabrier, M.A., Harris, J.W., Jeon, N.L., and Cotman, C.W. (2011). β-Amyloid impairs axonal BDNF retrograde trafficking. Neurobiol. Aging *32*, 821–833.

36. Kruttgen, A., Saxena, S., Evangelopoulos, M.E., and Weis, J. (2003). Neurotrophins and neurodegenerative diseases: receptors stuck in traffic? J. Neuropathol. Exp. Neurol. *62*, 340–350.

37. Millecamps, S., and Julien, J.P. (2013). Axonal transport deficits and neurodegenerative diseases. Nat. Rev. Neurosci. *14*, 161–176.

38. Chen, C., Wang, Z., Zhang, Z., Liu, X., Kang, S.S., Zhang, Y., and Ye, K. (2018). The prodrug of 7,8-dihydroxyflavone development and therapeutic efficacy for treating Alzheimer's disease. Proc. Natl. Acad. Sci. USA *115*, 578–583.

39. English, A.W., Liu, K., Nicolini, J.M., Mulligan, A.M., and Ye, K. (2013). Small-molecule trkB agonists promote axon regeneration in cut peripheral nerves. Proc. Natl. Acad. Sci. USA *110*, 16217–16222.

40. Liu, X., Erikson, C., and Brun, A. (1996). Cortical synaptic changes and gliosis in normal aging, Alzheimer's disease and frontal lobe degeneration. Dementia *7*, 128–134.

41. Terry, R.D., Masliah, E., Salmon, D.P., Butters, N., DeTeresa, R., Hill, R., Hansen, L.A., and Katzman, R. (1991). Physical basis of cognitive alterations in Alzheimer's disease: synapse loss is the major correlate of cognitive impairment. Ann. Neurol. *30*, 572–580.

42. Price, J.L., Ko, A.I., Wade, M.J., Tsou, S.K., McKeel, D.W., and Morris, J.C. (2001). Neuron number in the entorhinal cortex and CA1 in preclinical Alzheimer disease. Arch. Neurol. *58*, 1395–1402.

43. Cirrito, J.R., Yamada, K.A., Finn, M.B., Sloviter, R.S., Bales, K.R., May, P.C., Schoepp, D.D., Paul, S.M., Mennerick, S., and Holtzman, D.M. (2005). Synaptic activity regulates interstitial fluid amyloid-beta levels in vivo. Neuron *48*, 913–922.

44. Quartey, M.O., Nyarko, J.N.K., Maley, J.M., Barnes, J.R., Bolanos, M.A.C., Heistad, R.M., Knudsen, K.J., Pennington, P.R., Buttigieg, J., De Carvalho, C.E., et al. (2021). The Aβ(1-38) peptide is a negative regulator of the Aβ(1-42) peptide implicated in Alzheimer disease progression. Sci. Rep. *11*, 431.

45. DeKosky, S.T., and Scheff, S.W. (1990). Synapse loss in frontal cortex biopsies in Alzheimer's disease: correlation with cognitive severity. Ann. Neurol. *27*, 457–464.

46. Scheff, S.W., Sparks, D.L., and Price, D.A. (1996). Quantitative assessment of synaptic density in the outer molecular layer of the hippocampal dentate gyrus in Alzheimer's disease. Dementia *7*, 226–232.

47. Hattori, C., Asai, M., Onishi, H., Sasagawa, N., Hashimoto, Y., Saido, T.C., Maruyama, K., Mizutani, S., and Ishiura, S. (2006). BACE1 interacts with lipid raft proteins. J. Neurosci. Res. *84*, 912–917.

48. Kang, M.J., Chung, Y.H., Hwang, C.I., Murata, M., Fujimoto, T., Mook-Jung, I.H., Cha, C.I., and Park, W.Y. (2006). Caveolin-1 upregulation in senescent neurons alters amyloid precursor protein processing. Exp. Mol. Med. *38*, 126–133.

49. Domínguez-Prieto, M., Velasco, A., Tabernero, A., and Medina, J.M. (2018). Endocytosis and Transcytosis of Amyloid-β Peptides by Astrocytes: A Possible Mechanism for Amyloid-β Clearance in Alzheimer's Disease. J. Alzheimers Dis. *65*, 1109–1124.

50. Saavedra, L., Mohamed, A., Ma, V., Kar, S., and de Chaves, E.P. (2007). Internalization of beta-amyloid peptide by primary neurons in the absence of apolipoprotein E. J. Biol. Chem. *282*, 35722–35732.

51. Karataeva, A.R., Klaassen, R.V., Ströder, J., Ruiperez-Alonso, M., Hjorth, J.J., van Nierop, P., Spijker, S., Mansvelder, H.D., and Smit, A.B. (2014). C-terminal interactors of the AMPA receptor auxiliary subunit Shisa9. PLoS ONE *9*, e87360.

52. Khodosevich, K., Jacobi, E., Farrow, P., Schulmann, A., Rusu, A., Zhang, L., Sprengel, R., Monyer, H., and von Engelhardt, J. (2014). Coexpressed auxiliary subunits exhibit distinct modulatory profiles on AMPA receptor function. Neuron *83*, 601–615.

53. Kunde, S.A., Rademacher, N., Zieger, H., and Shoichet, S.A. (2017). Protein kinase C regulates AMPA receptor auxiliary protein Shisa9/CKAMP44 through interactions with neuronal scaffold PICK1. FEBS Open Bio *7*, 1234–1245.

54. Gaudreault, S.B., Dea, D., and Poirier, J. (2004). Increased caveolin-1 expression in Alzheimer's disease brain. Neurobiol. Aging *25*, 753–759.

55. Ikezu, T., Ueda, H., Trapp, B.D., Nishiyama, K., Sha, J.F., Volonte, D., Galbiati, F., Byrd, A.L., Bassell, G., Serizawa, H., et al. (1998). Affinity-purification and characterization of caveolins from the brain: differential expression of caveolin-1, -2, and -3 in brain endothelial and astroglial cell types. Brain Res. *804*, 177–192.

56. Niesman, I.R., Zemke, N., Fridolfsson, H.N., Haushalter, K.J., Levy, K., Grove, A., Schnoor, R., Finley, J.C., Patel, P.M., Roth, D.M., et al. (2013). Caveolin isoform switching as a molecular, structural, and metabolic regulator of microglia. Mol. Cell. Neurosci. *56*, 283–297.

57. Takeuchi, S., Matsuda, W., Tooyama, I., and Yasuhara, O. (2013). Kainic acid induces expression of caveolin-1 in activated microglia in rat brain. Folia Histochem. Cytobiol. *51*, 25–30.

58. Glass, C.K., Saijo, K., Winner, B., Marchetto, M.C., and Gage, F.H. (2010). Mechanisms underlying inflammation in neurodegeneration. Cell *140*, 918–934.

59. Head, B.P., Peart, J.N., Panneerselvam, M., Yokoyama, T., Pearn, M.L., Niesman, I.R., Bonds, J.A., Schilling, J.M., Miyanohara, A., Headrick, J., et al. (2010). Loss of caveolin-1 accelerates neurodegeneration and aging. PLoS ONE *5*, e15697.

60. Niesman, I.R., Schilling, J.M., Shapiro, L.A., Kellerhals, S.E., Bonds, J.A., Kleschevnikov, A.M., Cui, W., Voong, A., Krajewski, S., Ali, S.S., et al. (2014). Traumatic brain injury enhances neuroinflammation and lesion volume in caveolin deficient mice. J. Neuroinflammation *11*, 39.

61. Head, B.P., Patel, H.H., Tsutsumi, Y.M., Hu, Y., Mejia, T., Mora, R.C., Insel, P.A., Roth, D.M., Drummond, J.C., and Patel, P.M. (2008). Caveolin-1 expression is essential for N-methyl-D-aspartate receptor-mediated Src and extracellular signal-regulated kinase 1/2 activation and protection of primary neurons from ischemic cell death. FASEB J. *22*, 828–840.

62. Borchelt, D.R., Ratovitski, T., van Lare, J., Lee, M.K., Gonzales, V., Jenkins, N.A., Copeland, N.G., Price, D.L., and Sisodia, S.S. (1997). Accelerated amyloid deposition in the brains of transgenic mice coexpressing mutant presenilin 1 and amyloid precursor proteins. Neuron *19*, 939–945.

**OMTM, Volume *21***

## Supplemental information

# Synapsin-caveolin-1 gene therapy preserves neuronal and synaptic morphology and prevents neurodegeneration in a mouse model of AD

**Shanshan Wang, Joseph S. Leem, Sonia Podvin, Vivian Hook, Natalia Kleschevnikov, Paul Savchenko, Mehul Dhanani, Kimberly Zhou, Isabella C. Kelly, Tong Zhang, Atsushi Miyanohara, Phuong Nguyen, Alexander Kleschevnikov, Steve L. Wagner, John Q. Trojanowski, David M. Roth, Hemal H. Patel, Piyush M. Patel, and Brian P. Head**

**Supplementary Materials**

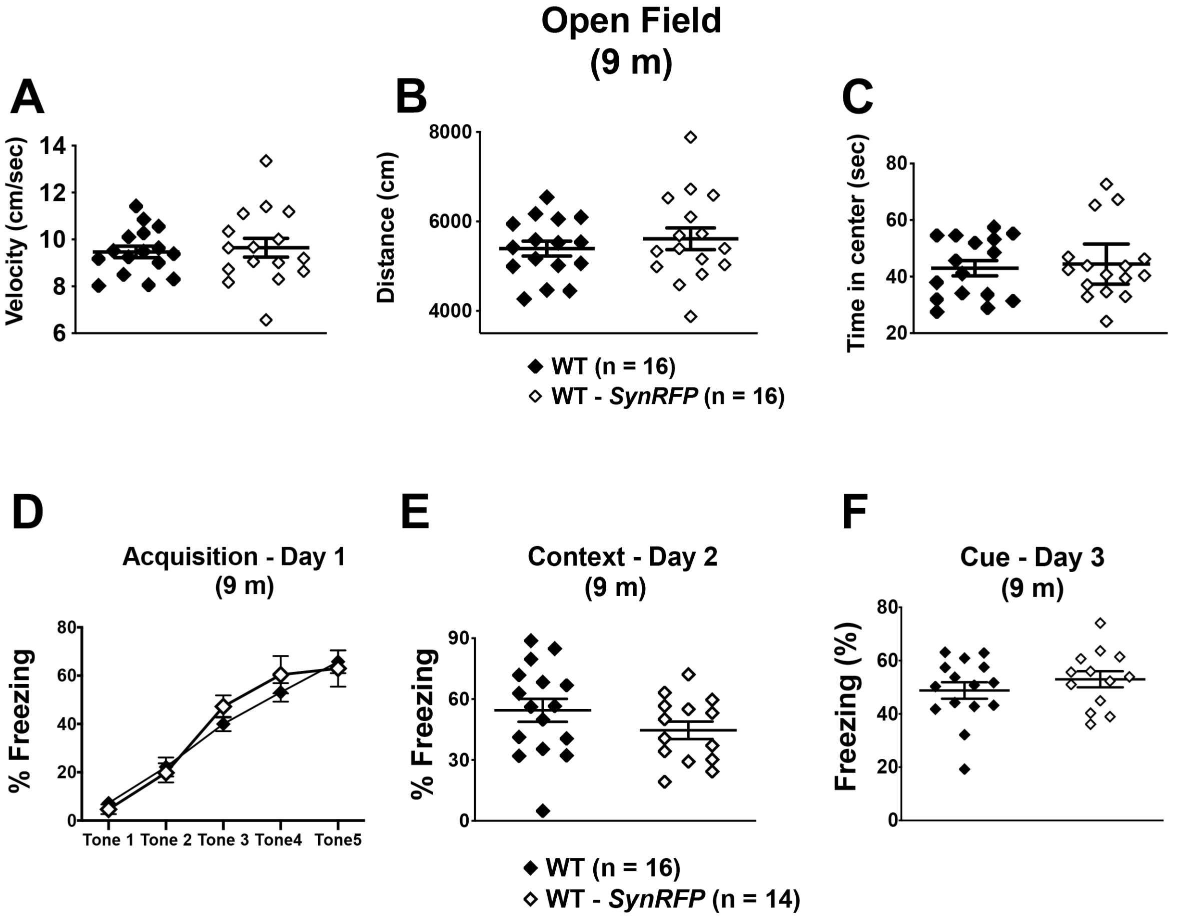


**Supplemental Figure 1** Open field and fear conditioning test comparing naïve WT with WT-*SynRFP* at 9 m. (**A-C**) Open field (velocity, distance moved, time in center) and (**D-F**) fear conditioning for naïve WT ($n$ = 16) versus WT-*SynRFP* ($n$ =14-16) at 9 m. Open field data (mean ± SEM) (percent (%) freezing mean ± SEM) were analyzed using Student $t$ test (Day 2 and 3). Significance was assumed when $p < 0.05$.

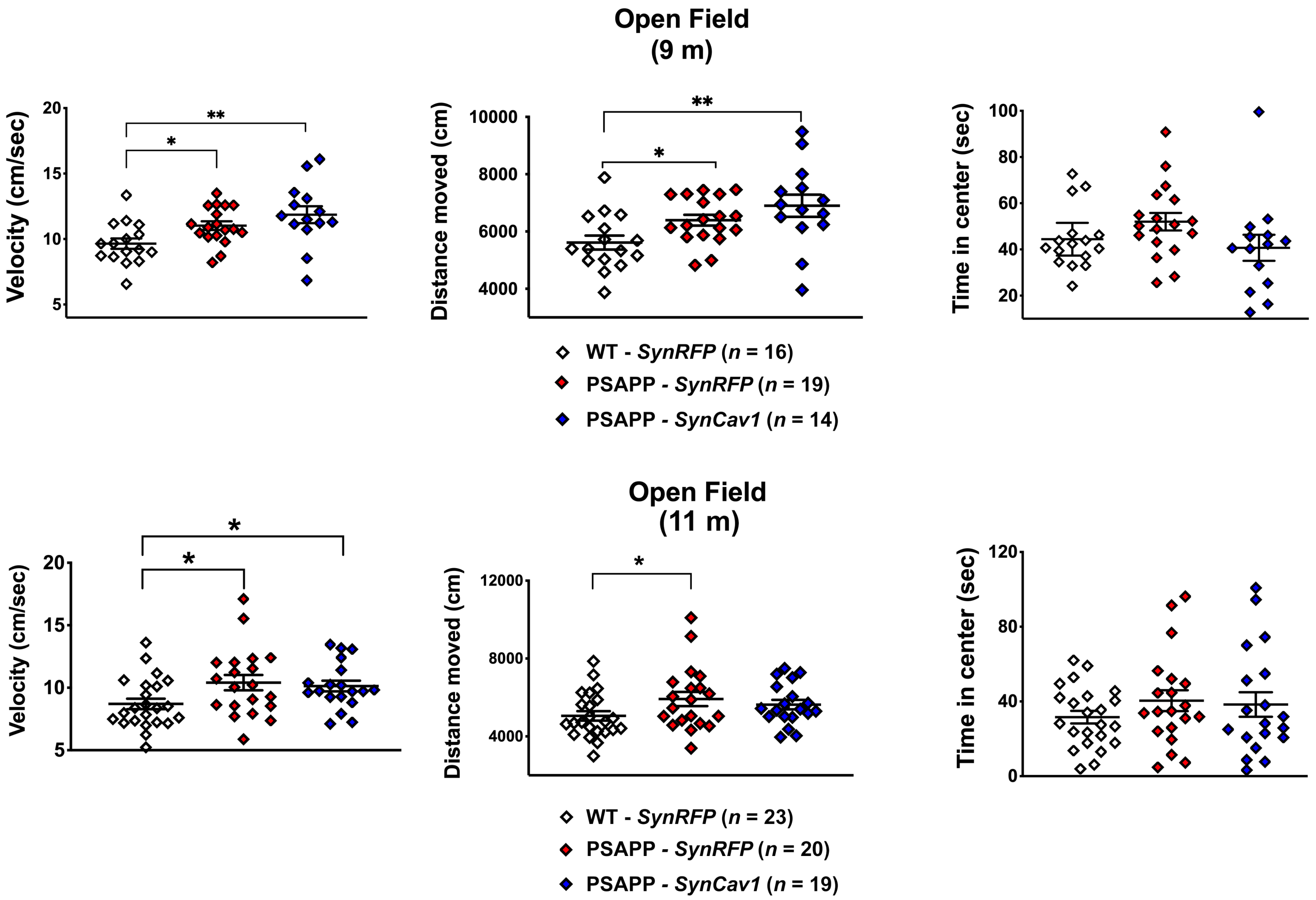


**Supplemental Figure 2** Open field performance of 9 and 11 m PSAPP *(APPSwePS1d9*) mice. (A, **D**) Mean velocity, (**B, E**) distance traveled, and (**C, F**) time spent in the center of 9 and 11 m old WT-*SynRFP* ($n$ = 16 at 9 m; $n$ = 23 at 11 m), PSAPP-*SynRFP* ($n$ = 19 at 9 m; $n$ = 20 at 11 m), and PSAPP-*SynCav1* ($n$ = 14 at 9 m; $n$ = 19 at 11 m) mice respectively. For open field data (mean ± SEM) was analyzed using one-way analysis of variance (ANOVA) followed by Fisher's LSD multiple comparisons tests. Data are presented as mean ± SEM. Significance was assumed when $p < 0.05$. *$p < 0.05$, **$p < 0.01$.

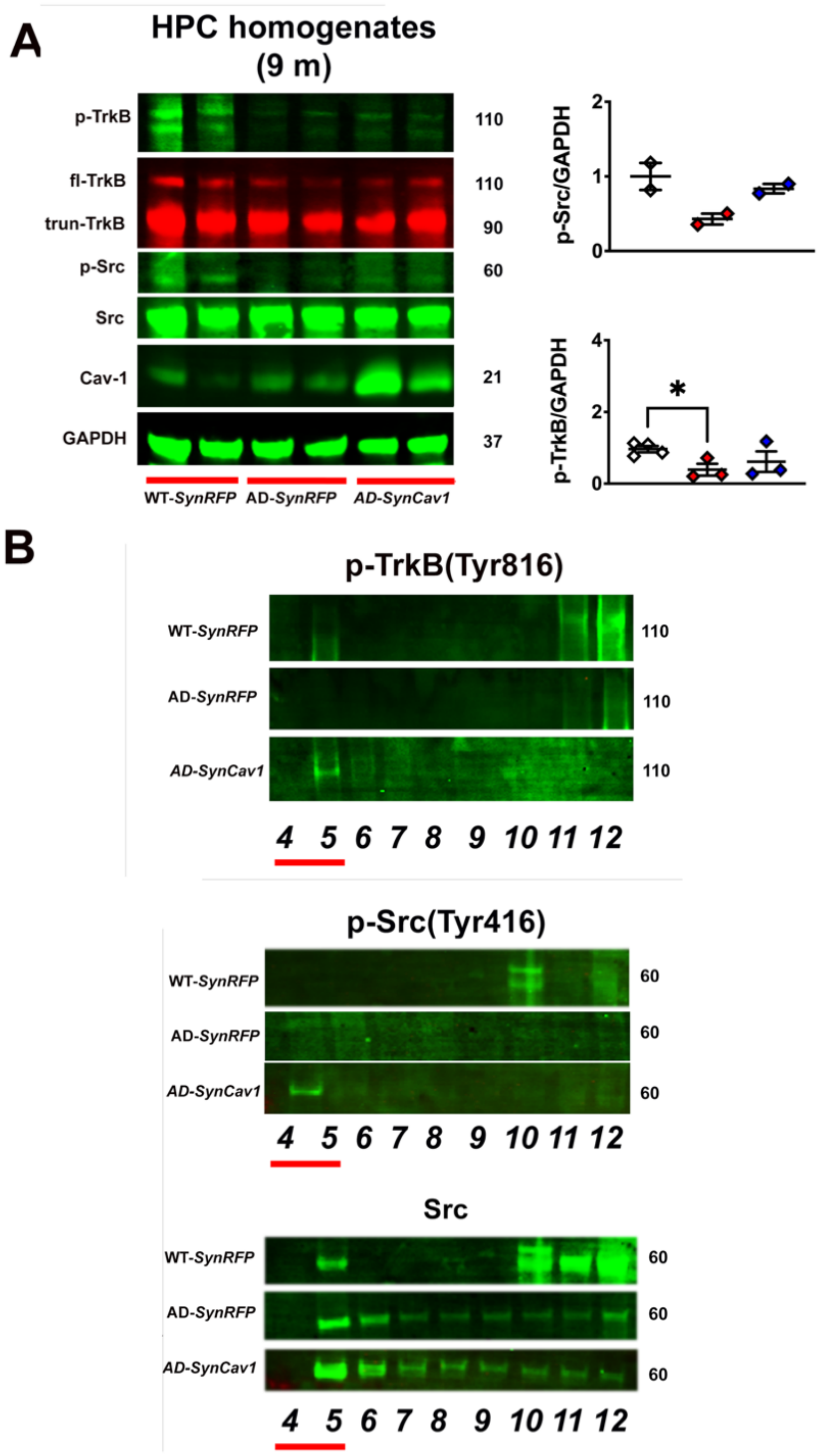


**Supplemental Figure 3** Assessment of TrkB activation in hippocampal homogenates and MLR fractions at 9 m. (**A**) Hippocampal homogenates from 9 m old WT-*SynRFP,* AD-*SynRFP*, and AD-*SynCav1* mice (n=3) were assayed for phosphorylated (p-)TrkB (Y816), full length (fl) and truncated (trun) TrkB, p-Src (Y416), Src, Cav-1, and GAPDH. (**B**) Fractions were assayed for p-TrkB (Y816) and p-Src (Y418). Fractions were generated

from equal protein (0.5 ug/ul). Data (mean ± SEM) were analyzed using one-way ANOVA. Significance was assumed when $*p < 0.05$.

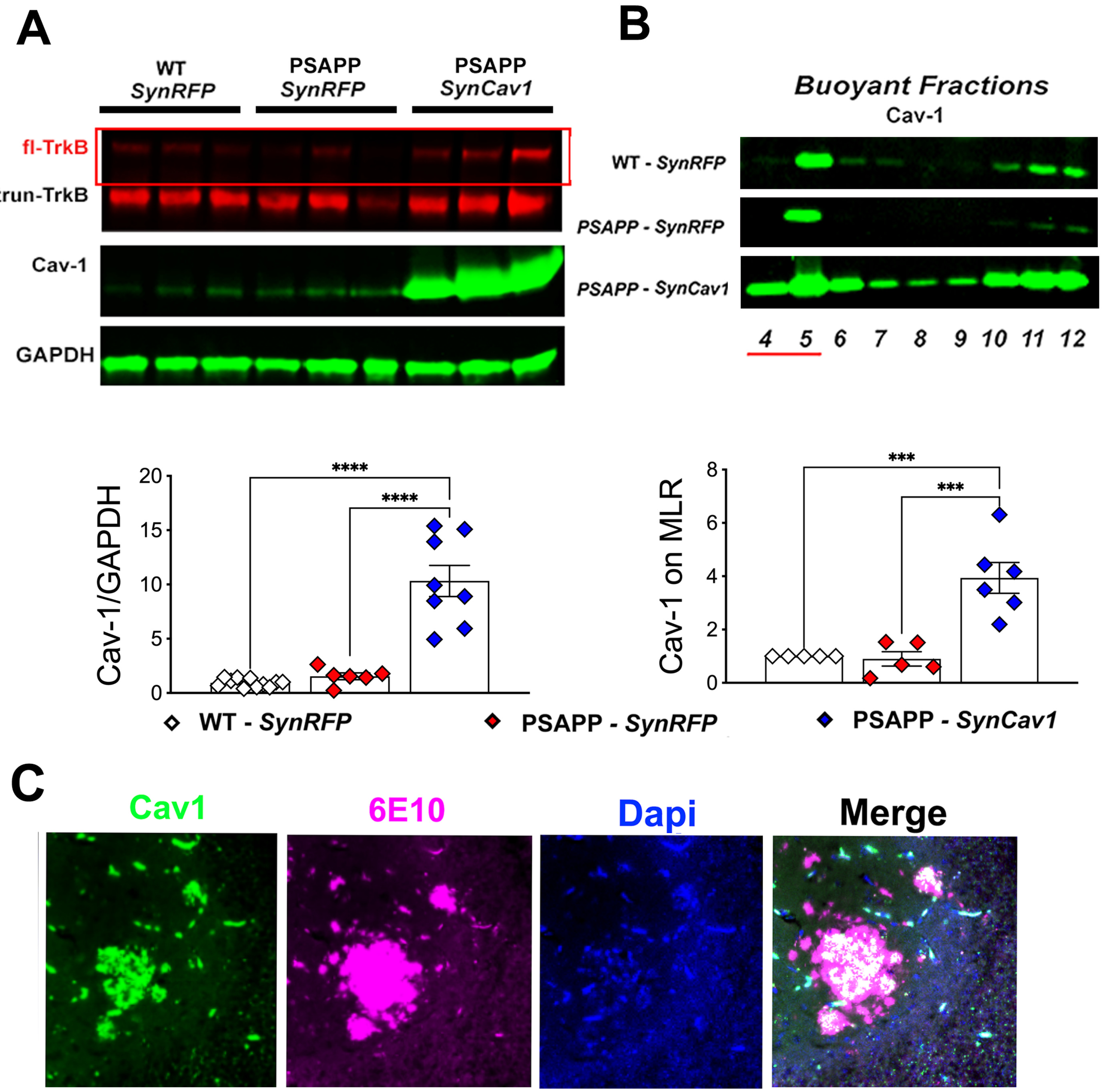


**Supplemental Figure 4 (A)** IB of HPC homogenates and **(B)** MLR fractions confirm the sustained overexpression of Cav1 in mice hippocampi. **(C)** Colocalization of Cav-1 and 6E10 was observed in 11-month

old PSAPP-SynRFP mice. Data (mean ± SEM) were analyzed using one-way ANOVA followed by Fisher's LSD multiple comparisons test. Significance was assumed when *$p < 0.05$.

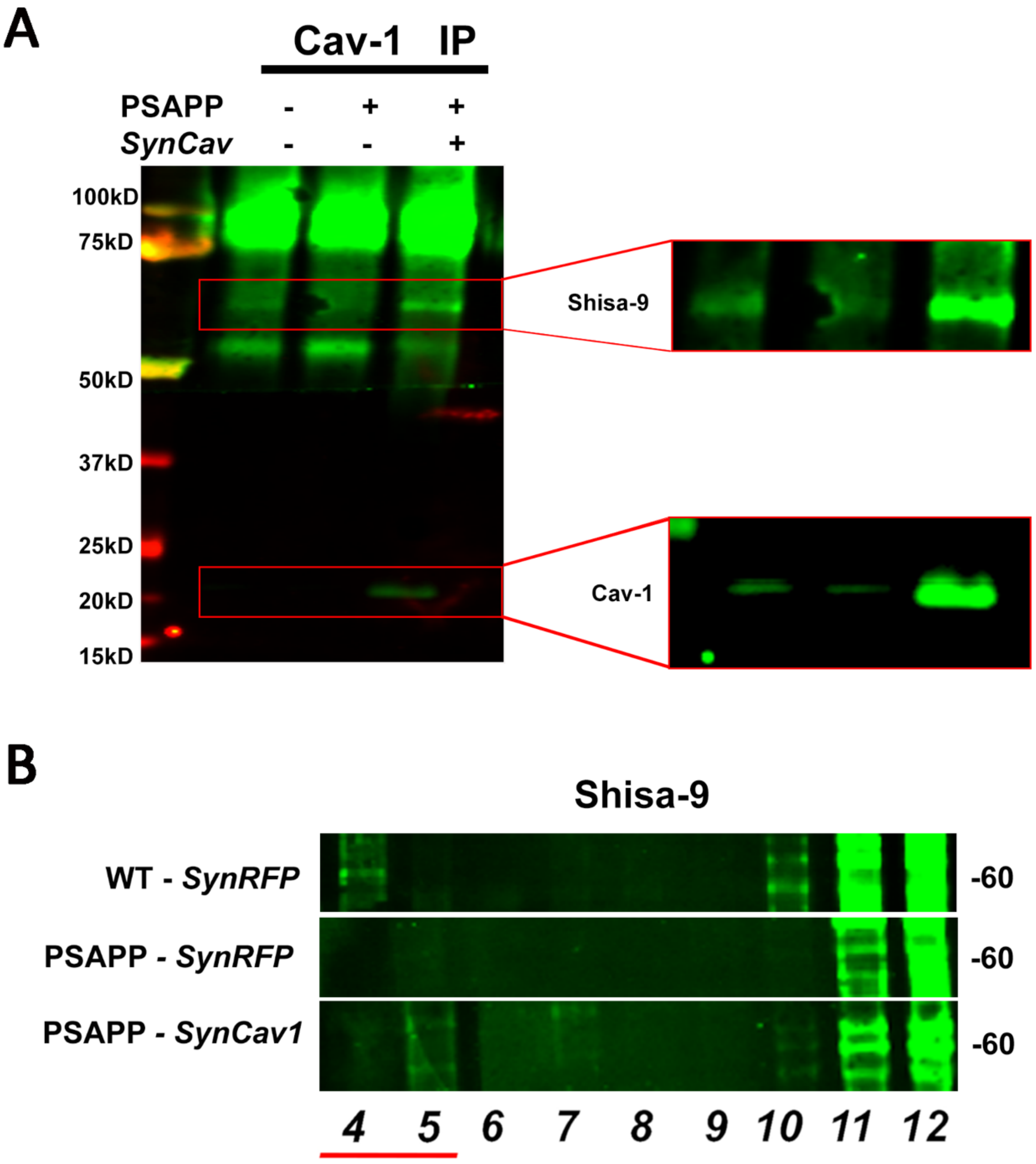


**Supplemental Figure 5** Cav-1 IPs of MLR fractions confirm the interaction of Shisa9 and Cav1 in mice hippocampi **(A)**. Cav-1 IP of MLR fractions revealed decreased Shisa9 expression in PSAPP -SynRFP and the highest Shisa9 expression in PSAPP-*SynCav1*. **(B)** Representative blot of Shisa9 in hippocampal MLR fractions in WT-*SynRFP*, PSAPP-*SynRFP*, and PSAPP-*SynCav1* mice at 9 m.

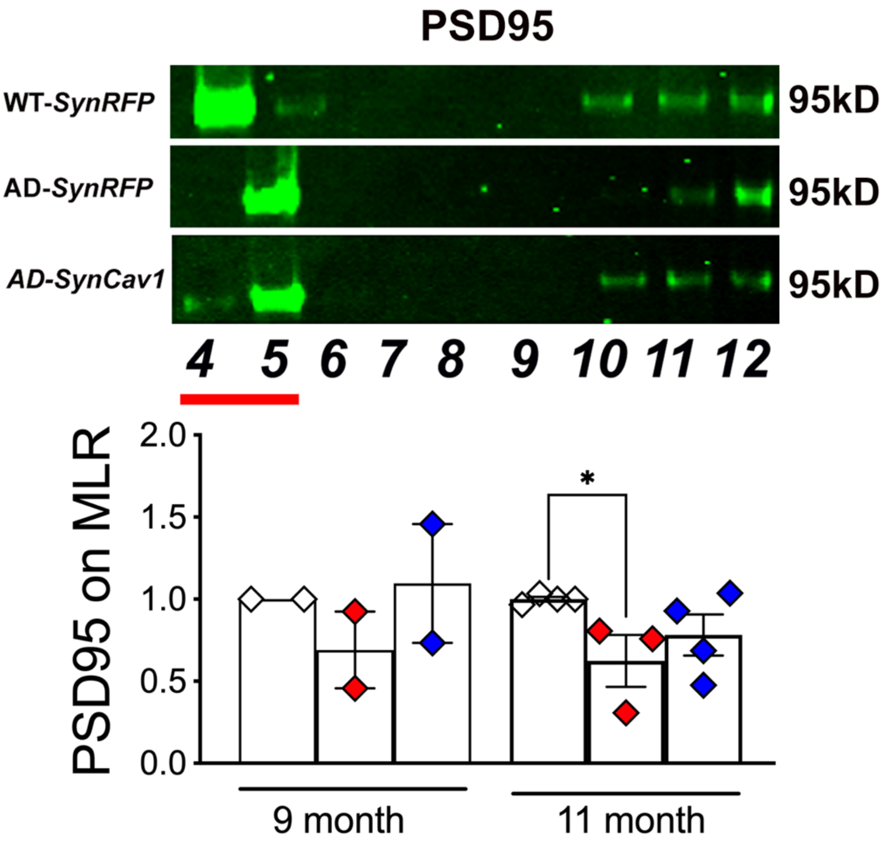


**Supplemental Figure 6** Immunoblots of MLR fractions revealed a decrease of PSD in 11 month PSAPP-*SynRFP*. Immunoblot is representative of 11-month samples. No significant difference was detected between AD-*SynCav1* and WT-*SynRFP* mice at 11 m.

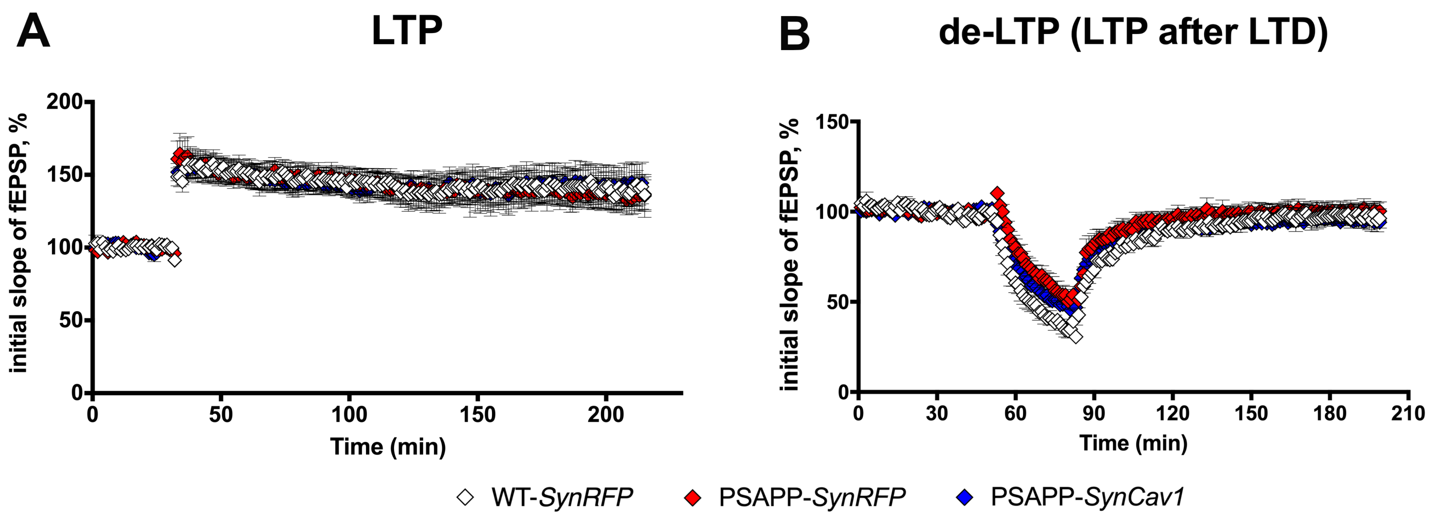


**Supplemental Figure 7** Electrophysiology reveals no significant difference in long-term potentiation (LTP) between WT-*SynRFP*, PSAPP-*SynRFP*, and PSAPP-*SynCav1* hippocampal slices (**A**). De-LTP (LTD after prior induction of LTP) was evoked by low-frequency stimulation (LFS, 15 min x 1 Hz) applied 3 hours after induction of LTP. The short-term depression (1-5 min) revealed no differences among the groups. Long-term changes (1-2 hours after LFS) were also not different among the groups. Data (mean ± SEM) were analyzed using two-way analysis of variance (ANOVA), *n* = 7-8 slices from 3-4 mice/group.

**Supplementary Video. 1** Light Sheet Microscopy AAV9-*SynRFP* injected mouse hippocampus (1-week post-injection), optically cleared in X-CLARITY hydrogel solution (Cat #13103). Data processing and 3D rendering was done using Arivis Vision4D™. Scale bar = 100 um
